# Synthetic Naphthoquinone Inhibits Herpes Simplex Virus Type-1 Replication Targeting Na^+^, K^+^ ATPase

**DOI:** 10.1021/acsomega.4c05904

**Published:** 2024-08-16

**Authors:** Kauê Francisco Corrêa de Souza e Souza, Vitor Won-Held Rabelo, Paula Alvarez Abreu, Cláudio César Cirne Santos, Nayane Abreu do Amaral e Silva, Daniela de Luna, Vitor Francisco Ferreira, Bernardo Ferreira Braz, Ricardo Erthal Santelli, Cassiano Felippe Gonçalves-de-Albuquerque, Izabel Christina Nunes de Palmer Paixão, Patricia Burth

**Affiliations:** † Departamento de Biologia Celular e Molecular, Instituto de Biologia, Universidade Federal Fluminense, Niterói, Rio de Janeiro CEP 24020-201, Brazil; ‡ Instituto de Biodiversidade e Sustentabilidade, Universidade Federal do Rio de Janeiro, Macaé, Rio de Janeiro CEP 27965-045, Brazil; § Departamento de Química, Instituto de Química, Laboratório de Catálise e Síntese (Lab CSI), Universidade Federal Fluminense, Niterói, Rio de Janeiro CEP 24020-141, Brazil; ∥ Departamento de Tecnologia Farmacêutica, Universidade Federal Fluminense, Faculdade de Farmácia, Niterói, Rio de Janeiro 24241-002, Brazil; ⊥ Departamento de Química Analítica, Instituto de Química, Universidade Federal do Rio de Janeiro, Rio de Janeiro, Rio de Janeiro CEP 21941-909, Brazil; # Laboratório de Imunofarmacologia, Instituto Oswaldo Cruz, FIOCRUZ, Rio de Janeiro, Rio de Janeiro CEP 21040-900 Brazil; ¶ Laboratório de Imunofarmacologia, Universidade Federal do Estado do Rio de Janeiro, Rio de Janeiro, Rio de Janeiro CEP 20211-010 Brazil

## Abstract

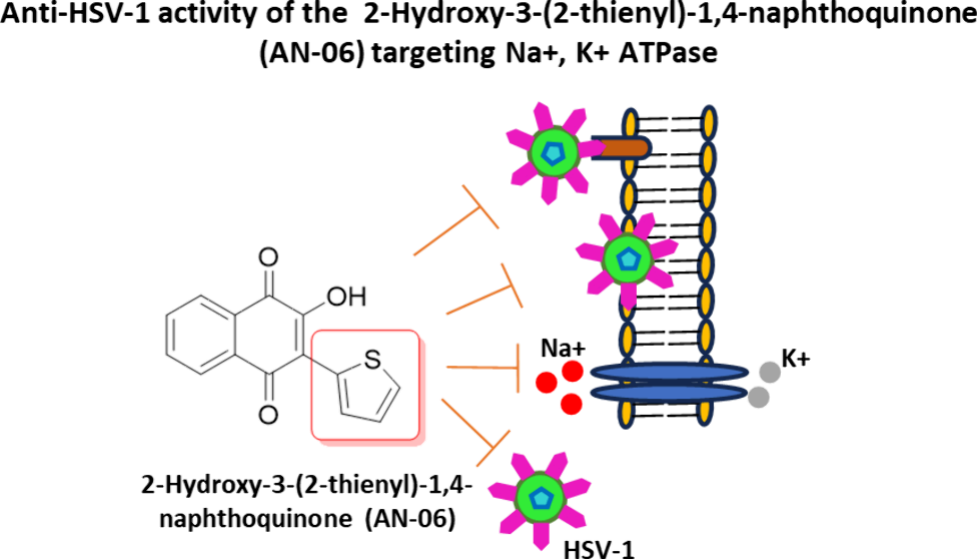

Since 1970 acyclovir (ACV) has been the reference drug in treating herpes simplex virus (HSV) infections. However, resistant herpes simplex virus type 1 (HSV-1) strains have emerged, narrowing the treatment efficacy. The antiviral activity of classical Na^+^, K^+^ ATPase enzyme (NKA) inhibitors linked the viral replication to the NKA’s activity. Herein, we evaluated the anti-HSV-1 activity of synthetic naphthoquinones, correlating their antiviral activity with NKA inhibition. We tested seven synthetic naphthoquinones initially at 50 μM on HSV-1-infected African green monkey kidney cells (VERO cells). Only one compound, 2-hydroxy-3-(2-thienyl)-1,4-naphthoquinone (AN-06), exhibited higher antiviral activity with a low cytotoxicity. AN-06 reduced the viral titer of 9 (log10) to 1.32 (log10) and decreased the steps of attachment and penetration. The addition of AN-06 up to 20 h postinfection (hpi) interfered with the viral cycle. The viral infection alone increases NKA activity 3 h postinfection (hpi), scaling up to 6 hpi. The addition of AN-06 in a culture infected with HSV-1 decreased NKA activity, suggesting that its antiviral action is linked to NKA inhibition. Also, docking results showed that this compound binds at the same site of NKA in which adenosine triphosphate (ATP) binds. AN-06 exhibited promising pharmacokinetic and toxicology properties. Thus, we postulate that AN-06 may be a good candidate for antiviral compounds with a mechanism of action targeting NKA activity.

## 1 Introduction

Herpes simplex viruses type-1 (HSV-1) are pathogens of global distribution with no specific seasonal variation, age, and sex^1^ and represent one of the most common infectious diseases in the population.^2^ The HSV-1 infection can unleash a productive disease, which may result in other pathological conditions. Some examples are mucocutaneous infections, encephalitis, keratoconjunctivitis (a leading cause of blindness in underdeveloped countries),^3,4^ genital infection,^5^ Alzheimer’s disease development^6^ and HSV lytic cycle reactivation in lung of patients infected with SARS-CoV-2.^7^

Since 1970, acyclovir (ACV) is the most effective antiviral for HSV-1 treatment.^8,9^ However, the appearance of HSV-1 strains resistant to ACV, has intensified the search for new molecules with potential antiviral properties and different mechanisms of action.^8,10^

Changes in ion influx in host cells infected by different viruses were reported a long time ago.^11−14^ In this context, inhibitors of Na^+^, K^+^ ATPase (NKA) (e.g., digoxin, digitoxin, digitoxigenin, ouabain, lanatoside C, cymarin, neriifoline, and strophanthin) which bind to the enzyme blocking it in the phosphorylated E2 conformational state^15^ have also shown antiviral activity.^16−24^ A review from our group discussed the importance of ion influx in host cells during viral infection proposing a relationship between NKA activity and alphavirus replication.^25^ NKA has been described to be important to viral proteins synthesis, virus release, viral cell to cell spread, mRNA expression,^26^ HSV-1 DNA synthesis, early and late gene expression.^27^

Natural drugs or synthesized from natural products have always been used in the medical clinic,^28^ where naphthoquinones represent a crucial group of compounds.^29^ Studies with its derivatives have shown their broad spectrum of action.^30−32^ For instance, they have an antiviral effect against cytomegalovirus (CMV),^33^ poliovirus type 2 (PV2),^34^ human immunodeficiency virus (HIV)^35^ and chikungunya virus (CHIKV).^36^ Herein, we evaluated seven synthetic naphthoquinones as anti-HSV-1 and correlated them with activity on NKA.

## 2 Materials and Methods

### 2.1 Cell Culture, Virus, and Compounds

African green monkey kidney (VERO) cell lineages, obtained initially from American type culture collection (ATCC) (CCL81) were cultured with Dulbecco’s modified Eagle medium (DMEM) (Invitrogen) supplemented with 10% heat-inactivated fetal bovine serum (FBS) (Invitrogen), antibiotics (100 U/mL Penicillin and 100 U/mL Streptomycin-Invitrogen), NaHCO_3_ (2.25 g/L) at 37 °C in a humid 5% CO_2_ atmosphere 5%. HSV-1 (KOS strain, ACV susceptible) was grown and propagated in VERO cells. Virus stocks were stored at −80 °C and titrated before use by plaque assay, as described by Cheng et al.^37^

**Figure 1:**
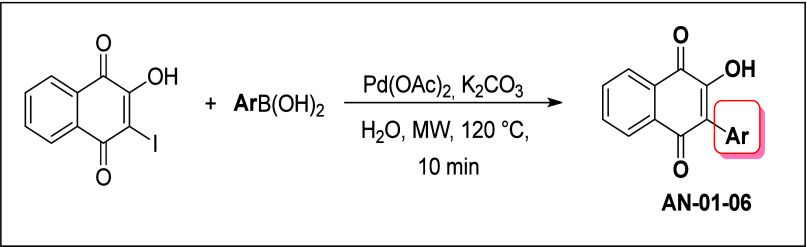
Scheme of Suzuki reaction between 2-hydroxy-3-iodo-1,4-naphthoquinone, and arylboronic acids.

The seven synthetic naphthoquinones tested are depicted in Table 1 and nuclear magnetic resonance spectroscopy (NMR spectra) in Figures S1–S19. The synthesis of 3-halogen-2-hydroxy-1,4-naphthoquinones (AN-02 and AN-03) was carried out from the reaction between the commercial lawsone (AN-01), and the halogens bromine and iodine. The 3-aryl-2-hydroxy-1,4-naphthoquinones (AN-04, AN-05, AN-06, and AN-07) were synthesized by Suzuki couplings^38,39^ between the 2-hydroxy-3-iodo-1,4-naphthoquinone (AN-03) and different arylboronic acids under basic conditions catalyzed by palladium (Figure 1). For all assays, the synthetic naphthoquinones and ACV were diluted in dimethyl sulfoxide (DMSO) 100% (SIGMA) and stored at −20 °C. Before each test, this stock solution (50 mM) was diluted in DMEM medium without FBS.

**Table 1:**
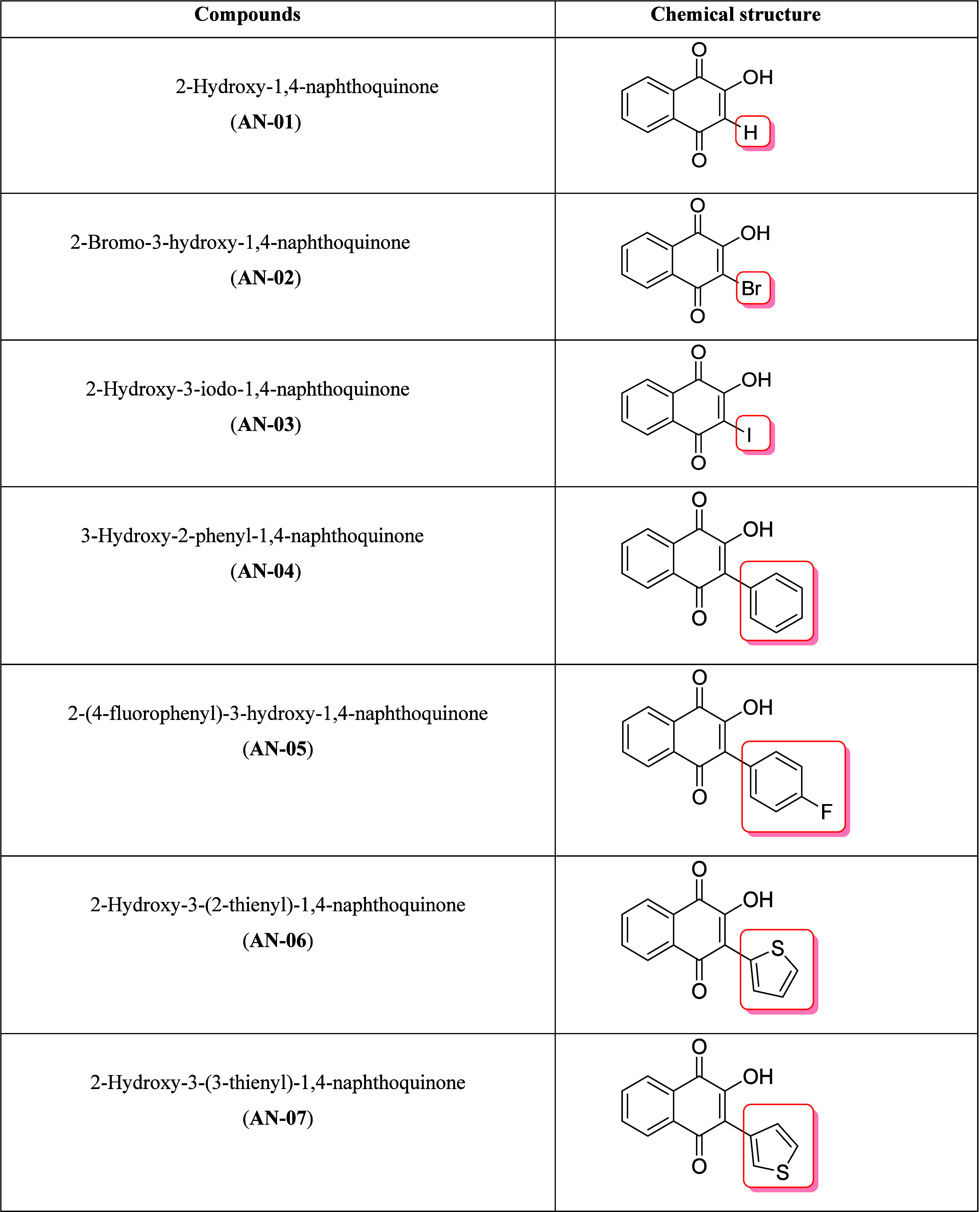
Chemical Structure of Synthetic Naphthoquinones

### 2.2 Plaque Reduction Assay

The plaque reduction assay followed the methodology described by Cheng et al.,^40^ with some modifications. VERO cells were infected with the HSV-1 KOS strain at a multiplicity of infection (MOI) of 1. After 1 h of incubation at 37 °C, the supernatant containing the viral inoculum was removed, and synthetic naphthoquinones were added at a concentration of 50 μΜ in the overlay medium (DMEM 2X, 5% fetal bovine serum and 2% of methylcellulose). After 72 h of incubation at 37 °C, the supernatant was collected in microtubes and stored at −80 °C. Another plate with VERO cell monolayers maintained in 24-multiwell was done. The samples collected were diluted and incubated on cell monolayers for 1 h. At the end 72 h, the 24-multiwell plates were fixed and stained with a solution containing 0,1% crystal violet and 10% formalin. As control for the maximum antiviral activity, cells were treated with ACV 50 μM and diluted in DMEM medium without FBS. The antiviral activity was determined by the difference between treated samples and DMSO 0,1% samples.

### 2.3 Prediction of Pharmacokinetics and Toxicological Properties of the Naphthoquinone Derivatives

To investigate the drug-like profile of the naphthoquinone derivatives, the theoretical pharmacokinetic and toxicological (ADMET) properties of these compounds were calculated and compared with ACV. The human intestinal absorption (HIA) and the ability of the compounds to bypass the blood-brain barrier (BBB) were predicted using the admetSAR 2.0 server.^41^ The FAF-Drugs4 server^42^ was employed to calculate the stereochemical properties of the compounds and evaluate them by applying rules developed by pharmaceutical industries such as Lipinski “rule of five”, GlaxoSmithKline (GSK) 4/400 and Pfizer 3/75. The first one states that compounds with good oral bioavailability have to meet, at least, three of the following features: molecular weight (MW) ≤ 500 Da; LogP ≤ 5; the number of hydrogen bond acceptor (HBA) and donor (HBD) groups ≤10 and 5, respectively.^43^ The GSK 4/400 rule defines that compounds with MW > 400 Da and LogP > 4 are more likely to present ADMET issues^44^ while the Pfizer 3/75 rule indicates that compounds with LogP > 3 and topological polar surface area (tPSA) < 75 Å^2^ are more frequently associated with toxicity events in animal models like dogs and rats.^45^

The cardiotoxic effect was analyzed by the ability of the compound to inhibit both potassium ion channel human ether-a-go-go-related gene (hERG) I and II using the pkCSM server.^46^ The mutagenicity, tumorigenicity, and irritant toxicity risks, as well as the drug-score, were evaluated using the Osiris Property Explorer server (https://www.organic-chemistry.org/prog/peo/).

### 2.4 Cytotoxicity Assay

The cytotoxic effect of synthetic naphthoquinone AN-06 was evaluated by colorimetric quantification of cell death based on the determination of the activity of lactate dehydrogenase (LDH) released into the supernatant. VERO cells were treated with different concentrations (50, 100, 250, 500, 1000, 2000, and 4000 μM) of AN-06. After 72 h of incubation, the supernatant was collected, and the LDH activity was measured on a spectrophotometer at 510 nm. As a control for the maximum LDH release, cells were treated with 0.1% Triton-X100 in DMEM medium without FBS for 30 min before running the assay. The cell viability in control samples (DMSO 0.1%) was 100%, and the number of viable cells was determined by the difference between the percentage of treated samples and the DMSO 0.1% sample. The colorimetric quantification of released LDH was analyzed by a DOLES kit.

### 2.5 Virucidal Assay

The virucidal activity was evaluated according to the methodology described by Schuhmacher et al.^47^ with some modifications. HSV-1 suspensions, containing 1 × 10^6^ plaque-forming units (P.F.U) mL^–1^, were diluted in the presence or absence of synthetic naphthoquinone AN-06 (50 μΜ) or DMSO 0.1% or ACV (50 μΜ) and stored at room temperature for 1 h. Next, treated or untreated viral suspensions were centrifuged, resuspended in DMEM medium without FBS, and added on VERO cell monolayers maintained in 24-multiwell plates for 1 h at 37 °C. At the end of 1 h, the virus inoculum was removed, and cells were covered with the overlay medium and incubated for 72 h. DMSO 0.1% was used as a negative control. The residual infectivity was determined by plaque assay, as described by Cheng et al.^37^

### 2.6 Attachment and Penetration Assay

The attachment assay followed the methodology described by Gong et al.^48^ with some modifications. VERO cells were prechilled at 4 °C for 1 h, and then infected with HSV-1 at a MOI of 0.0003 in the presence or absence of the AN-06 at 50 μM or acyclovir at 50 μΜ and incubated for 1 h at 4 °C. Unabsorbed viruses were removed by washing with cold phosphate buffer solution 1× (PBS 1×), the cell monolayers were covered with overlay media and incubated for 72 h at 37 °C. The attachment inhibition percentage was analyzed by plaque assay, as described by Cheng et al.^37^

For penetration assay, we followed the methodology described by Rosenthal et al.^49^ with some modifications. VERO cells were infected with HSV-1, MOI of 0.0003, for 1 h at 4 °C. After cooling, the cells were washed with PBS 1×, treated or not with AN-06 at 50 μΜ or ACV at 50 μΜ, and incubated at 37 °C for 1 h. Posteriorly, the unpenetrated viruses were treated with PBS (pH 3) for 1 min and immediately neutralized with PBS (pH 11) for 1 min. Then, the cells were covered with overlay media and incubated at 37 °C for 72 h. The penetration inhibition percentage was analyzed by plaque assay, as described by Cheng et al.^37^

### 2.7 Time-of-Addition assay

The effects of AN-06 were evaluated during alpha, beta, and gamma phases of HSV-1 replication, according to the methodology described by Gong et al.^48^ with some modifications. VERO cells were infected with HSV-1, MOI of 0.0003, for 1 h at 37 °C. The virus inoculum was removed, and fresh media with AN-06 (50 μΜ) was added at different times according to the replication phases: 0 to 3 h post infection (hpi) (alpha phase), 3 to 6 hpi (beta phase), and 6 to 20 hpi (gamma phase). At the end of each phase, the supernatant was removed, and cells were washed with PBS 1× followed by the addition of overlay media and incubation for 72 h at 37 °C. The inhibition percentage of each phase was analyzed by plaque assay, as described by Cheng et al.^37^

### 2.8 Na^+^, K^+^ ATPase Activity Assay

NKA activity of VERO cells infected with HSV-1, MOI of 0.1, was determined according to the methodology described by Gill et al.^50^ After infection, the monolayers cell were washed with PBS 1x and treated with Hank’s solution (NaCl 136.9 mM, MgSO_4_ 0.8 mM, NaHCO_3_ 5 mM, Na_2_HPO_4_ 0.33 mM, NaH_2_PO_4_ 0.44 mM, CaCl_2_ 1.5 mM, Glucose 3 mM, Hepes (2-[4–92-hydroxyethyl)piperazin-1-yl]ethanesulfonic 5 mM) in the presence or absence of AN-06 at 50 μΜ or ACV at 50 μΜ and incubated at 37 °C with 5% CO_2_ in time points 3, 6, 9, and 20 hpi. At the end of each time, the cell monolayers were washed with PBS 1× and Hank’s solution with Rb^+^ (NaCl 136.9 mM, MgSO_4_ 0.8 mM, NaHCO_3_ 5 mM, Na_2_HPO_4_ 0.33 mM, NaH_2_PO_4_ 0.44 mM, CaCl_2_ 1.5 mM, Glucose 3 mM, Hepes 5 mM e RbCl 5.4 mM) with or without ouabain 0.5 mM and incubated at 37 °C for 30 min. After 30 min of incubation, the cell monolayers were lysed with sodium dodecyl sulfate (SDS) 0.15%, stored in microtubes, and Rb^+^ was quantified by inductively coupled plasma mass spectrometry (ICP-MS).

### 2.9 Homology Modeling

Since the naphthoquinone derivatives are planned to be drug candidates for human use, the molecular modeling studies were carried out using the human NKA. The human NKA sequence was retrieved from the National Center for Biotechnology Information (NCBI) database under the accession code CAA31390.1. The three-dimensional (3D) structure of the human NKA was constructed using homology modeling with the software Modeler v. 9.24.^51^ The enzyme was modeled in two different conformational states, E1 and E2. For the NKA model in the E1 form, the NKA from pig kidney (*Sus scrofa*) in complex with adenosine diphophate (ADP) (PDB code 3WGU)^52^ was chosen as the template. The NKA from shark (*Squalus acanthias*) complexed with ouabain (PDB code 3A3Y)^53^ was used as the template for the E2 state model.

Following models’ construction, their quality was assessed by a stereochemical analysis using the Ramachandran plot generated with Procheck,^54^ analysis of the compatibility between the primary and tertiary structures using the average 3D-1D score calculated with Verify-3D^55^ and analysis of the overall energy profile based on the Z-score calculated using the ProSA-web server.^56^

### 2.10 Molecular Docking and Structural Comparison with Known NKA Inhibitors

The structural similarity of AN-06 and inhibitors of NKA was carried out based on the Tanimoto coefficient (Tc), which ranges from 0 to 1 (1 corresponding to identical structures). The two-dimensional structures of AN-06, known human NKA inhibitors available in the ChEMBL database (345 compounds) and nonsteroidal NKA inhibitors reviewed by Alevizopoulos and co-workers^57^ were compared employing the marginal abatement cost curves (MACCS) fingerprint with the Open Babel 2.4.1 software.^58^

The 3D structure of the most potent compound AN-06 and the known NKA inhibitors ouabain and quercetin was constructed and optimized using Spartan’ 10 software (Wave function Inc. Irvine, CA, USA). Initially, a conformational search was carried out in vacuum using molecular mechanics and the merck molecular force field

(MMFF) force field. Subsequently, the lowest-energy conformer was subjected to geometry optimization employing the semiempirical method (RM1), followed by ab initio calculation using the density functional theory (DFT) method with B3LYP/6-31G* basis set. The final structure was used as an input for the docking studies.

To validate the docking method, we first carried out a redocking of the ouabain within its binding site in the shark NKA in the E2 form protein data bank (PDB) code 3A3Y, using the software autodock tools 1.5.7 (ADT) and autodock 4.2.6.^59^ The grid box was centered on the CB atom of T804 (equivalent to the T796 in the human NKA), and its dimensions were set to 60 × 64 × 64 points with a grid spacing of 0.375 Å. A similar approach was undertaken to validate the docking method regarding the adenosine triphosphate (ATP) binding site within the E1 state of the NKA. The ADP was redocked in the pig NKA (PDB code 3WGU). The grid box dimensions were defined as 40 × 46 × 72 points (0.375 Å spacing), and it was centered on the CA atom of G502 (equivalent to the G501 in the human NKA). For both cases, the Lamarckian genetic algorithm was chosen as the search engine, and 50 poses were calculated. The other parameters were kept as a default. After validation, these protocols were employed for the docking studies with the human NKA in the E1 and E2 states. The binding mode and protein–ligand interaction analysis were carried out using Discovery Studio Visualizer 2019 (Dassault Systèmes BIOVIA, San Diego, 2019) and Pymol version 1.2r2 (The PyMOL Molecular Graphics System, Version 1.2r2 Schrödinger, LLC).

### 2.11 Statistical Analysis

Prism 5.0 software (GraphPadInc., CA, USA) was used for graphical presentation and statistical analysis. The statistical analyses included Student’s *t* test and one-way ANOVA followed by the Bonferroni test. The data are expressed as the means ± standard deviation of at least three independent experiments. Significance was determined at *p < 0.05; **p < 0.01; ***p < 0.001.

## 3 Results

### 3.1 Antiviral Activity and Cytotoxicity Evaluation

The first step was to test the antiviral activity of the synthesized compounds. We tested seven synthetic naphthoquinones at the initial concentration of 50 μΜ for antiviral activity on VERO cells treated for 72 h. Only one compound (AN-06) exhibited good antiviral activity, inhibiting 54% of PFU, athough it presented lower activity than ACV (97.66%). The concentration that inhibits 50% of PFU (EC_50_) of AN-06 and ACV was calculated as (39.12 ± 6) and (1.42 ± 0.61), respectively. When AN-06 was added at 50, 100, and 250 μΜ, cell death reached 30% while in the presence of ACV at 50 μΜ, cell death was nearly 15%. The AN-06 and ACV cytotoxicities were greater than 50% in concentrations above 1000 μΜ. At the end, the selectivity index (SI) was also calculated, 19.4 for AN-06 and 660.56 for ACV. Nonetheless, these results were statistically different from the ones obtained for cells treated with Triton X100 (Table 2).

**Table 2:** Table Displaying Averaged CC_50_, EC_50_ and SI Values Obtained in This Study^a^

	HSV-1		VERO	
Compounds	Inhibition (%)	EC_50_ (μM)	CC_50_ (μM)	SI
AN-1	2 ± 2	ND	ND	ND
AN-2	20.82 ± 0.69	ND	ND	ND
AN-3	31.15 ± 0.73	ND	ND	ND
AN-4	0.3 ± 0.57	ND	ND	ND
AN-5	0.0 ± 0.0	ND	ND	ND
AN-6	51.06 ± 5.28	39.12 ± 6	760.1 ± 1.1	19.4
AN-7	6.02 ± 5.7	ND	ND	ND
ACV	98	1.42 ± 0.6	938 ± 7.9	660.5

a EC_50_ - concentration that inhibits 50% of PFU. CC_50_ - concentration that reduces 50% of cell viability. ND - not determined. SI - selectivity index.

### 3.2 ADMET Profile of the Naphthoquinone Derivatives

We employed *in silico* tools and industry rules to evaluate the drug-like profile of the naphthoquinone derivatives comparing with the anti-HSV-1 drug ACV (Table 3). Initially, we observed that all derivatives are likely absorbed in the human intestine, like ACV. Considering these compounds for oral delivery, we also evaluated them based on the Lipinski “rule of five”. All compounds fulfill these criteria, suggesting that they probably have a good oral bioavailability. Besides, we analyzed whether the compounds could reach the central nervous system (CNS) because HSV-1 causes CNS manifestations and can establish latent infection in neurons. Our results suggest that AN-06 and AN-07 can bypass the BBB and reach the CNS like ACV.

**Table 3:** In Silico ADMET Parameters of the Naphthoquinone Derivatives (AN-01-AN-07) and the Anti-HSV-1 Drug ACV^a^

						Toxicity risks				
Compounds	HIA^b^	BBB^b^	Ro5	GSK 4/400	Pfizer 3/75	Mutagenic	Tumorigenic	Irritant	Cardiotoxicity	Drug-score
**AN-01**	+(0.99)	–(0.50)	Pass	Pass	Warning	High	Low	Low	No	0.32
**AN-02**	+(0.97)	–(0.34)	Pass	Pass	Warning	Low	Low	Low	No	0.43
**AN-03**	+(0.96)	–(0.34)	Pass	Pass	Warning	Low	Low	Low	No	0.48
**AN-04**	+(0.99)	–(0.66)	Pass	Pass	Warning	Low	Low	Low	No	0.53
**AN-05**	+(0.99)	–(0.32)	Pass	Pass	Warning	Low	Low	Low	No	0.46
**AN-06**	+(0.99)	+(0.91)	Pass	Pass	Pass	Low	Low	Low	No	0.66
**AN-07**	+(0.99)	+(0.87)	Pass	Pass	Pass	Low	Low	Low	No	0.50
**ACV**	+(0.97)	+(0.99)	Pass	Pass	Pass	Low	Low	Low	No	0.94

a HIA: human intestinal absorption. BBB: the ability of a compound to cross the blood-brain barrier. Ro5: Lipinski “rule of five”.

b Probability of classification accuracy by the predictive model is shown in parentheses.

Moreover, we analyzed these compounds based on the GSK 4/400 and Pfizer 3/75 rules. According to the GSK 4/400, all compounds were approved, and consequently, they are less likely to have pharmacokinetics and toxicological issues. On the other hand, compounds AN-01 and AN-05 received a warning based on the Pfizer 3/75 rule because of their low tPSA (values shown in Table S1). However, AN-06 and AN-07 were also approved like ACV, suggesting that these compounds have little chance of causing preclinical toxicity. To further explore their toxicological profile, we evaluated some toxicity risks, like mutagenicity, tumorigenicity, cardiotoxicity, and irritant effect. Interestingly, most of the compounds showed low toxicity for all risks assessed, except AN-01, which showed a high mutagenic risk. Finally, we calculated the drug-score values of these compounds. The drug-score values range from 0 to 1.0, and the higher this value, the more superior the potential of the compound as a drug candidate. Compound AN-06 showed the highest drug-score value (0.66) among the naphthoquinone derivatives studied, which points to the best profile as a drug candidate in this series. Thus, we use AN6 in the following experiments.

### 3.3 Antiviral Mechanism Evaluation of AN-06

#### 3.3.1 Virucidal Profile

**Figure 2:**
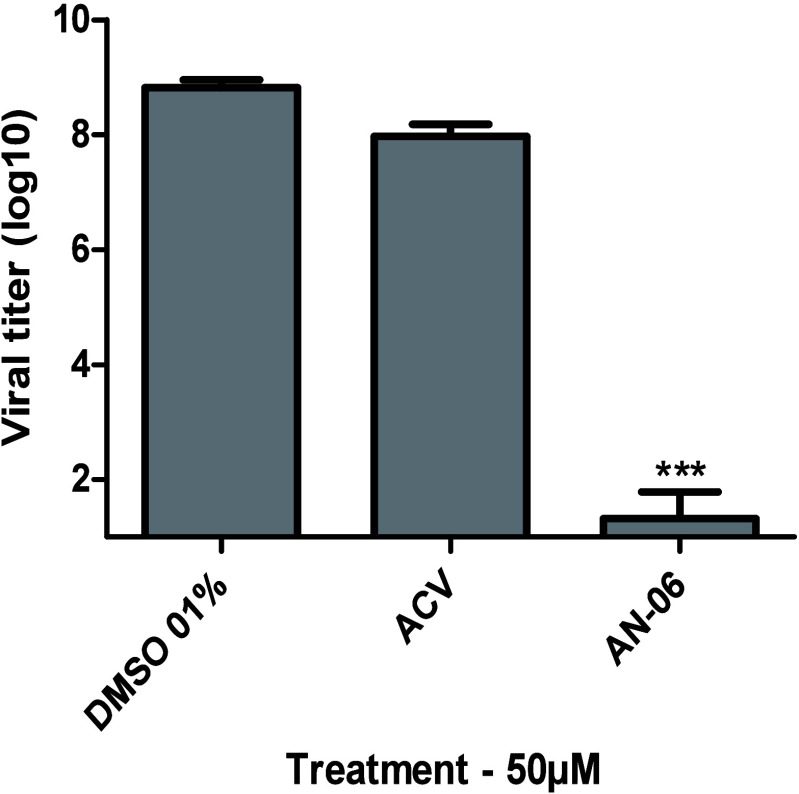
Virucidal effect of the naphthoquinone AN-06 in VERO cells infected with HSV-1 (KOS strain, MOI of 0.0003) for 72 h. The values represent the mean ± standard deviation of three independent experiments. *p < 0.05; **p < 0.01; ***p < 0.001 indicate significant statistical differences between DMSO 0.1% and treated samples.

To understand the mechanism of action of AN-06, we carried out an assay to test the effect of the compound on viral particles (virucidal assay). The preincubation of HSV-1 suspension with AN-06 for 1 h reduced the viral titer of 9 log_10_ to 1.32 log_10_ while ACV was able to reduce virus titer to 7.9 log_10_ (Figure 2).

#### 3.3.2 Attachment, Penetration, and Time-of-Addition Assay

**Figure 3:**
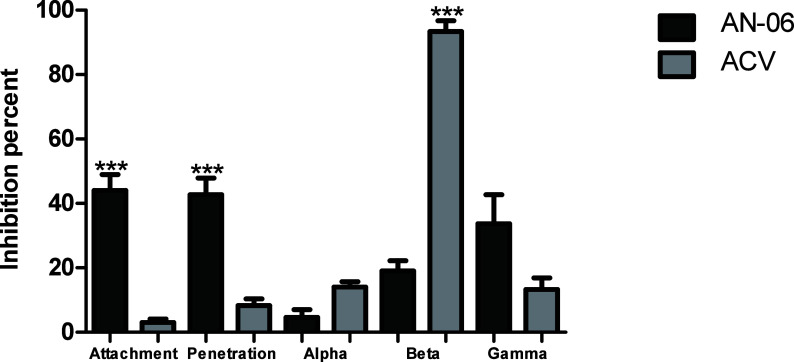
Effect of the naphthoquinone AN-06 (50 μM) at different stages of HSV-1 infection (KOS strain, MOI of 0.0003) on VERO cells for 72 h. Acyclovir (ACV; 50 μM) was used as a positive control. The values represent the mean ± standard deviation of three independent experiments. *p < 0.05; **p < 0.01; ***p < 0.001 indicate significant statistical differences between ACV-treated and AN-06-treated samples at each time point.

To further explore the mechanism by which AN-06 acts, we treated the infected cells at different moments during the HSV-1 replicative cycle. Compound AN-6 inhibited both virus attachment and penetration steps (50% inhibition; Figure 3). The time of addition of compound also were analyzed in specific time intervals (0 to 3 hpi - alpha, 3 hpi to 6 hpi - beta, and 6 hpi to 20 hpi - gamma steps). This compound did not affect virus replication when added to 3 and 6 hpi. By contrast, this compound exhibited higher inhibitory potential when added at the gamma phase. As expected, ACV showed remarkable inhibition (93.3%) when added at the beta phase (Figure 3).

### 3.4 NKA Activity of Cells Infected by HSV-1

**Figure 4:**
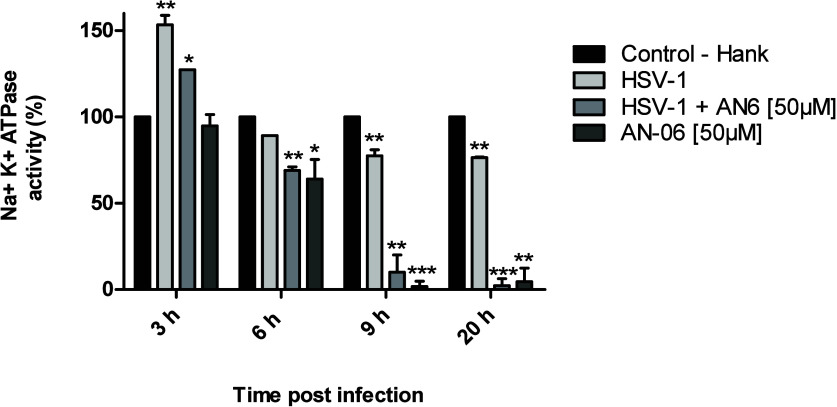
Effect of the naphthoquinone AN-06 (50 μM) on the Na^+^, K^+^ ATPase activity in VERO cells during infection by HSV-1 (KOS strain, MOI 0.1). The times (0–3 hpi, 3–6 hpi, 6–20 hpi) correspond, respectively, to the alpha, beta, and gamma phases of viral replication. The values represent the mean ± standard deviation of three independent experiments. *p < 0.05; **p < 0.01; ***p < 0.001 indicate significant statistical differences compared to the cell control treated with Hank solution.

As inhibitors of NKA present antiviral activity, we tested the effect of compound AN-06 on this enzyme’s function at different periods (0–3 hpi, 3–6 hpi, 6–9 hpi, 9–20 hpi) during HSV-1-infection (Figure 4).

We observed that HSV-1 infection increased by 50% of the enzyme activity at 3 hpi in comparison to mock-infected cells. At 6 hpi, the enzyme activity decreased and reached 77.6% at 20 hpi. However, the presence of AN-06 decreased the enzyme activity of HSV-1-infected cells at all times. Specifically, at 3 hpi, AN-06 did not reduce the enzymatic activity of the mock-infected cells. Also, we did not observe morphological changes in infected and treated cells, even at 20 hpi. (Figure S20).

### 3.5 Binding Mode of Compound AN-06 with the Human NAK Model

Since AN-06 was the best anti-HSV-1 compound and also inhibited the cellular target, NKA, we assessed the binding mode of this compound with the human NKA in the E1 and E2 states. According to the Ramachandran plot and Z-score of the constructed models, both E1 and E2 NKA showed good stereochemistry and overall energy profile (Table S2). Besides, the analysis of the 3D-1D score of their residues indicated that their sequences are compatible with the three-dimensional structures obtained. These results pointed to the excellent reliability of the models constructed and allowed their use in docking studies.

Initially, we validated our docking protocol by redocking ouabain and ADP within the shark NKA-E2 and pig NKA-E1 enzymes, respectively, which showed good prediction accuracy since we observed root-mean-square deviation (RMSD) values of 1.17 and 0.56 Å, respectively. Thereby, we employed these protocols to evaluate the binding manner of AN-06 with the human NKA in the E1 and E2 states. Our docking studies showed that AN-06 has a slightly higher theoretical affinity with the ATP binding site within the NKA-E1 state (binding energy = −6.01 kcal/mol) in comparison with the ouabain binding site of NKA-E2 (binding energy = −5.64 kcal/mol). AN-06 did not resemble the binding mode of ouabain in NKA-E2, which might be explained by their low similarity (Tc = 0.41) (Figure S2) and suggests that this compound does not as a classic NKA inhibitor. On the other hand, the structural comparison between AN-06 and other known inhibitors revealed that quercetin has the highest similarity with it (Tc = 0.57). Consequently, we focused our attention on the binding mode of AN-06 within the ATP binding site of the E1 state, which seems to be the likely mechanism of action, and compared it with quercetin, which is a proven NKA inhibitor.

**Figure 5:**
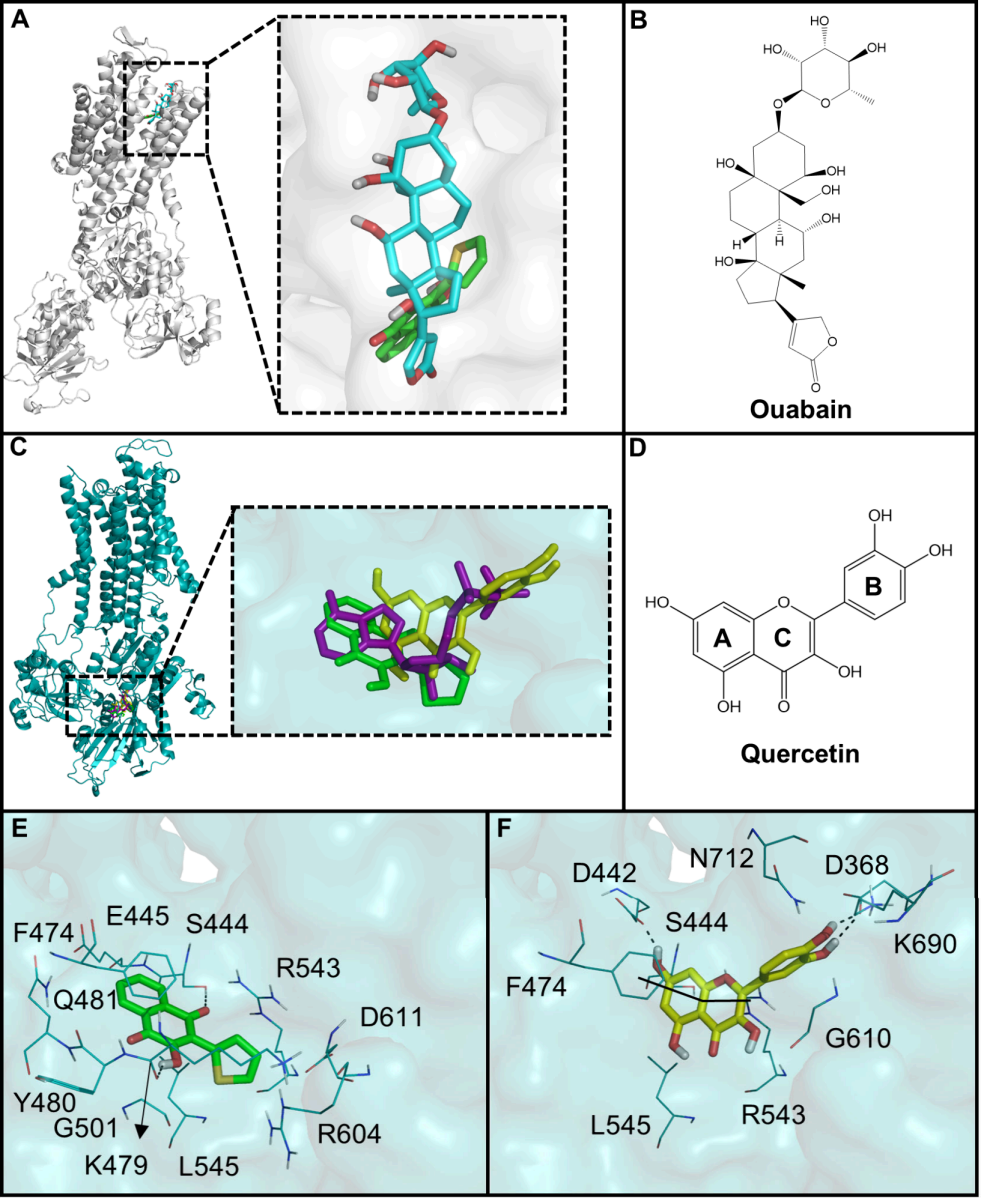
Molecular docking of AN-06 and known inhibitors with human NKA. (A) Superimposition of the AN6 (green) and ouabain (cyan) binding poses with human NKA in the E2 state and (B) chemical structure of ouabain. (C) Superimposition of the AN-06 (green) and quercetin (yellow) bound to the human NKA in the E1 state (NKA-E1) and ADP (purple) bound to the pig NKA-E1; (D) chemical structure of quercetin; and interaction network observed for (E) AN-06, and (F) quercetin. Hydrogen bonds are represented as dashed lines, while π–π stacking and cation-π interactions are shown as solid lines.

The binding mode of AN-06 at the ATP binding site showed that the naphthoquinone moiety was superimposed with the adenine nucleus of the ADP (Figure 5). As a result, hydrogen bond interactions were established between the 1,4-naphthoquinone core with S444 and K479. This group also interacted with E445, F474, Y480, Q481, K500, G501, and L545 through van der Waals interactions. The thiophene ring of AN-06 explored the same binding region as the sugar of ADP and was involved in van der Waals contacts with R543, D611, and R684. Quercetin showed a slightly different binding mode. However, it still explored the same region as the ADP, which is probably a consequence of its bulkier ring B in comparison with the thiophene ring of AN-06. Nonetheless, rings A and C of quercetin conserved similar contacts as observed for AN-06 (e.g., S444, F474, R543, and L545), whereas ring B bound at the same region as noticed for the phosphate groups of the ADP.

## 4 Discussion

In this study, we analyzed the antiviral activity of seven synthetic naphthoquinones, and the mechanism of action of the most potent compound was explored. Among the seven synthetic naphthoquinones tested, AN-06 presented an antiviral effect with a significant inhibition of viral plaques. Interestingly, AN-06 showed a low cytotoxic effect on VERO cells, even when added at concentrations ten times higher than the one used in the antiviral assays. A study also highlighted the relationship between antiviral activity and the radical linked to the naphthalene ring after analyzing HSV-1 and HSV-2 serotypes.^60^

Since poor pharmacokinetic and toxicological properties are the leading cause of drug failure in clinical trials,^61−63^ we also employed *in silico* tools to explore these properties of the naphthoquinone derivatives. Excitingly, the most potent compound AN-06 showed the most promising profile as a drug candidate with excellent intestinal absorption, oral bioavailability, and low toxicity risks, as pointed out by toxicity risk models and rules developed by pharmaceutical industries. Besides, AN-06 is likely able to bypass the blood-brain barrier (BBB) and can reach the CNS like the known antiherpetic drug ACV. The last characteristic is an attractive feature for anti-HSV1 drug candidates because HSV-1 causes CNS manifestations and can establish latent infection in neurons.

Different mechanisms may compose the antiviral activity. The interaction between HSV-1 and the compound test can change the viral structure and inactivate the particle permanently.^64^ Seeking to understand the mechanism of antiviral action of AN-06, we first tested whether this compound could interact with virus particles. The virucidal activity assay showed that the incubation of HSV-1 suspension with AN-06 for 1 h before infection reduced viral title of 9 (log10) to 1.32 (log10) indicating that AN-06 interacts with some viral component. Conversely, a study with 2-aminomethyl-3-hydroxy-1,4-naphthoquinone reported a virucidal activity of less than 50%.^65^ The noninactivation of viral particles by ACV in the virucidal assay was excepted because this compound acts on viral DNA polymerase during the beta step.^66^

The initial steps of viral infection are attachment and penetration. So, we analyzed the effects of AN-06 on these steps. We showed that AN-06 inhibited both the viral attachment and penetration, though the precise mechanism remains unknown because it may involve several glycoproteins and cellular proteins. However, it has been shown that cardiac glycosides (CGs) potently hamper the entry of the viral particles into cells. This process does not occur by blocking the NKA ionic pumping, but by SRC signaling pathway activation that triggers the transactivation of the PI3K/PDK1/RSK2 pathway of NKA. Also, the CG may block the entry of the virus into cells by via the NKA-independent JAK1 pathway.^67^ We also investigated the effects of AN-06 on further steps of viral replication. We showed that AN-06 presented viral inhibitory effects if added to up to 20 hpi. In this phase, the expression of genes that encode structural proteins of the capsid, tegument proteins, and envelope glycoproteins takes place, which are vital to assembly, maturation, and release of viral particles by exocytosis.^68−71^ However, further experiments would be necessary to verify which of these phases would be impaired. Despite this, our data show that AN-06 has an action mechanism to ACV.

For a strategy for the development of new antiherpes drugs, it is interesting to consider targeting crucial cellular proteins in different stages of HSV infection.^72^ Cellular proteins can also be considered as potential targets during the infection. Among cellular proteins, the NKA has been suggested as a critical factor^23^ for HSV-1 infection.^20,22^ The correlation between antiviral activity and the change in the NKA activity has been described.^22,73,74^

For instance, digitoxin and its structural analogs digoxin, ouabain, glucoevatromonoside, and G-strophanthin octahydrate showed higher antiviral activity against both wild-type and ACV-resistant HSV-1 and HSV-2 strains in comparison to ACV and ganciclovir.^21,22^ Glucoevatromonoside inhibited HSV-1 and HSV-2 replication at very low concentrations and blocked the viral proteins synthesis (ICP27, U_L_42, gB, gD) as well as virus particle release and cell-to-cell spread. Digitoxin interfered with the early and late gene expression and HSV-1 DNA synthesis in VERO cells. The same was observed during the release of viral particles that were gradually inhibited with increasing digoxin concentration.^27^ Ouabain inhibited transmissible gastroenteritis coronavirus decreasing the number of viral RNA copies.^75,76^ Also, ouabain and digitoxin inhibited viral mRNA expression, copy number, and viral protein expression of SARS-CoV-2 at the postentry stage.^26^ Hence, cardiac glycosides and other NKA inhibitors are promising compounds as antiviral agents.

Herein, we showed the potent effect of AN-06 on NKA activity at 6 and up to 20 hpi. The AN-06 addition during viral infection also decreased NKA activity at time 3 hpi, conversely gradually reducing NKA activity and its effect on times 6, 9, and 20 hpi, showing a growing inhibitory effect over the enzyme. AN6 alone, without virus infection, did not alter the NKA activity at time 3 h but inhibited enzyme function on times 6, 9, and 20 h. Also, we did not observe any cytopathic effect on cells cultivated up to 20 h.^77^ However, additional experiments in terms of treatment time have yet to be performed.

We also explored the mechanism of the inhibition of AN6 using computational approaches. Many nonsteroidal compounds have different mechanisms of inhibition of NKA compared with the cardiotonic steroids.^78−81^ Considering that different conformational forms of NKA can be found during ions transport,^82^ we modeled this protein in the E1 and E2 states and investigated the possible mechanism of action of AN-06 by docking studies. E1 and E2 states are NKA forms that present high affinity for sodium and potassium respectively and the transition from phosphorylated E1P to E2P is coupled to release of ADP, opening of the outer gate and release of the three sodium ions to the extracellular side.^83^ Our results suggested that AN-06 has a higher affinity with the ATP binding site of human NKA in the E1 state in comparison with the ouabain binding site in the NKA E2 state. In addition, AN-06 is structurally more similar to another compound (quercetin) than ouabain.

Quercetin and isoquercitrin are flavonoid with antiviral activity, interfering directly in viral entry.^84^ Quercetin inhibits ATP hydrolysis, prevents the formation of the E2 state of electric eel NKA,^78^ and was shown to compete with ATP binding in other ATPases.^85,86^ Interestingly, 1,4-naphthoquinone has been shown to inhibit the ATPase function of other enzymes and compete with ATP binding^87^ as well.^88^ AN-06 exhibited a similar binding manner and interaction pattern, as observed for the known inhibitor quercetin and the ADP bound to the pig NKA.^52^ Hence, our theoretical findings suggest that AN-06 inhibits NKA by binding to the ATP binding site, unlike the classical steroidal NKA inhibitors. Furthermore, our results can support further structural optimization of the naphthoquinone derivatives to improve their activity. For instance, the introduction of polar substituents attached to the thiophene ring or even its replacement by other polar moieties seems to be a reasonable approach since this group was positioned in a region rich in charged residues like R543, D611, and R684.

The HSV-1 replication in VERO cells seems to be dependent on the NKA activity. An increase in NKA activity at the early stages of viral infection has been reported,^74,89^ but further investigation remains to be done. Otherwise, the reduction of the NKA activity by HSV-1 from 6 hpi appears to be a viral mechanism to inhibit cellular protein synthesis.^90^ The peak of viral genes transcription occurs at 5–7 hpi, when proteins and enzymes needed for viral replication are encoded.^90,91^ The viral replication ends up in cytopathic effect, suggesting that the reduction of NKA activity (20 to 40%) could be linked to cellular protein synthesis.^92^ Nevertheless, further studies are still necessary to identify which viral proteins would be inhibited as well as factors that lead to increased NKA activity in the early stages of infection.

## 5 Conclusion

In this study, we evaluated the anti-HSV-1 activity of seven naphthoquinone derivatives. Among them, the compound showed the highest activity with low cytotoxicity. We observed that this compound interacts with HSV-1 particles, is effective during steps of attachment and penetration, and reduces the viral yield when added to up to 20 hpi. Also, this compound decreased NKA activity in infected cells, and docking simulations indicated that it binds at the ATP binding site, which supports this enzyme as a possible antiviral target in HSV-1 infections. Finally, compound AN-6 presented good theoretical pharmacokinetics and toxicological profiles comparable to those of the marketed drug ACV, reinforcing its potential as an anti-HSV-1 drug candidate.

## Supporting Information Available

^1^H and ^13^C nuclear magnetic resonance spectroscopy, infrared spectrum, physicochemical descriptors, homology modeling results for human Na^+^, K^+^ ATPase models in E1 and E2 states and their templates as well as illustrative images of the VERO cells infected and treated (PDF)
