# Supplementary material for: Synthetic Naphthoquinone Inhibits Herpes Simplex Virus Type-1 Replication Targeting Na+, K+ ATPase
Source: ACS Omega. 2024 Aug 16;9(34):36835–46. doi: 10.1021/acsomega.4c05904 (PMC11360054; doi:10.1021/acsomega.4c05904)
Supplement: Supplementary file 1 — ao4c05904_si_001.pdf [file ao4c05904_si_001.pdf]

Synthetic naphthoquinone inhibits herpes simplex virus type-1 replication targeting Na^+, K^+ ATPase

Kauê Francisco Corrêa de Souza e Souza[a], Vitor Won-Held Rabelo[a], Paula Alvarez Abreu[b], Cláudio César Cirne Santos[a], Nayane Abreu do Amaral e Silva[c], Daniela de Luna[c], Vitor Francisco Ferreira[d], Bernardo Ferreira Braz[e], Ricardo Erthal Santelli[e], Cassiano Felippe Gonçalves-de-Albuquerque[f,g], Izabel Christina Nunes de Palmer Paixão[a], Patricia Burth[a]

[a] Departamento de Biologia Celular e Molecular, Instituto de Biologia, Universidade Federal Fluminense, Niterói, RJ CEP 24020-201, Brazil.
[b] Instituto de Biodiversidade e Sustentabilidade, Universidade Federal do Rio de Janeiro, Macaé, RJ, CEP 27965-045, Brazil.
[c] Departamento de Química, Instituto de Química, Laboratório de Catálise e Síntese (Lab CSI), Universidade Federal Fluminense, Niterói, RJ, CEP 24020-141, Brazil.
[d] Departamento de Tecnologia Farmacêutica, Universidade Federal Fluminense, Faculdade de Farmácia, Niterói - RJ, 24241-002, Brazil.
[e] Departamento de Química Analítica, Instituto de Química, Universidade Federal do Rio de Janeiro, Rio de Janeiro, RJ, CEP 21941-909, Brazil.
[f] Laboratório de Imunofarmacologia, Instituto Oswaldo Cruz, FIOCRUZ, Rio de Janeiro, RJ CEP 21040-900 Brazil.
[g] Laboratório de Imunofarmacologia, Universidade Federal do Estado do Rio de Janeiro, Rio de Janeiro, RJ CEP 20211-010 Brazil.
Correspondence Kauê Francisco Corrêa de Souza e Souza: kauefcorrea@hotmail.com

AN-01

2-hydroxy-1,4-naphthoquinone (lawsone or 2-hydroxy-naphthalen-1,4-dione)

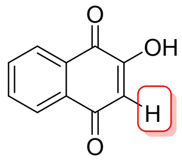

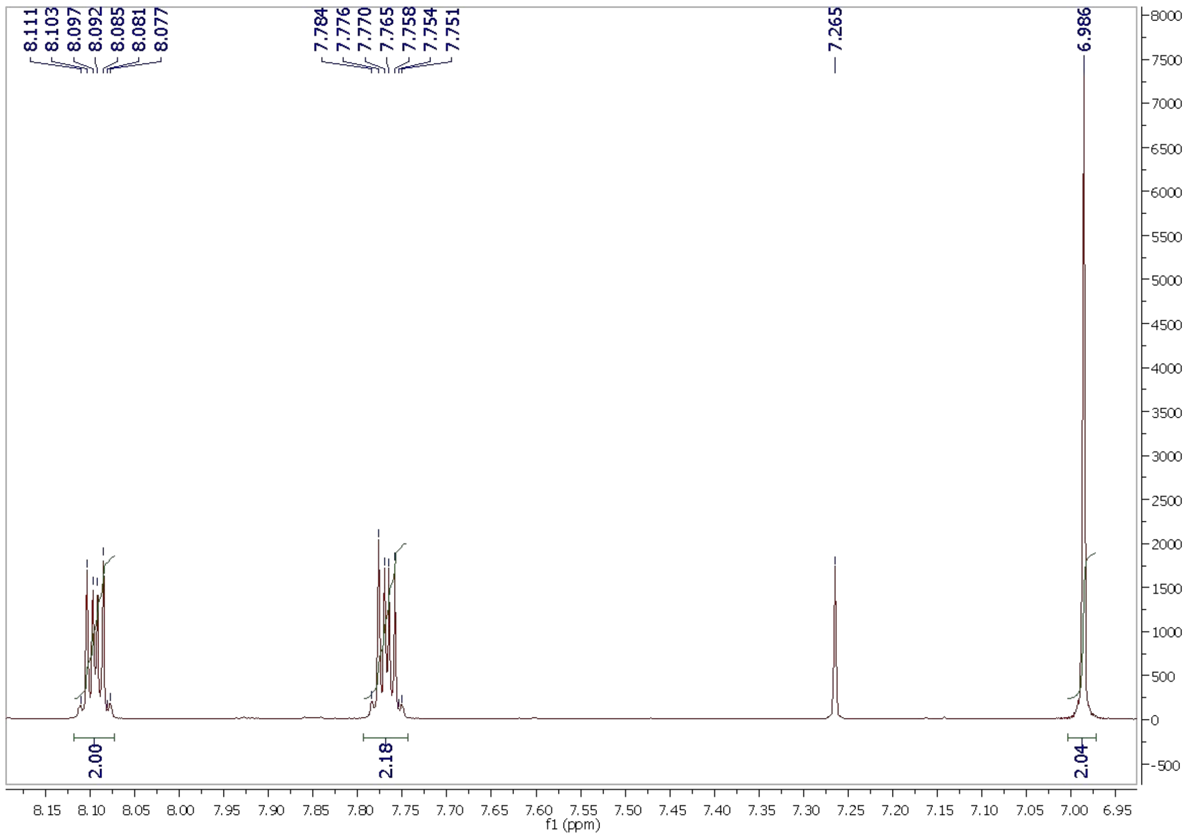

Figure S1 - ^{1}H-NMR spectrum of a commercial 2-hydroxy-1,4-naphthoquinone (**AN-01**)

AN-02

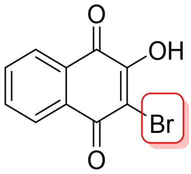

2-bromo-3-hydroxy-1,4-naphthoquinone (2-bromolawsone or 2-bromo-3-hydroxynaphthalen-1,4-dione)

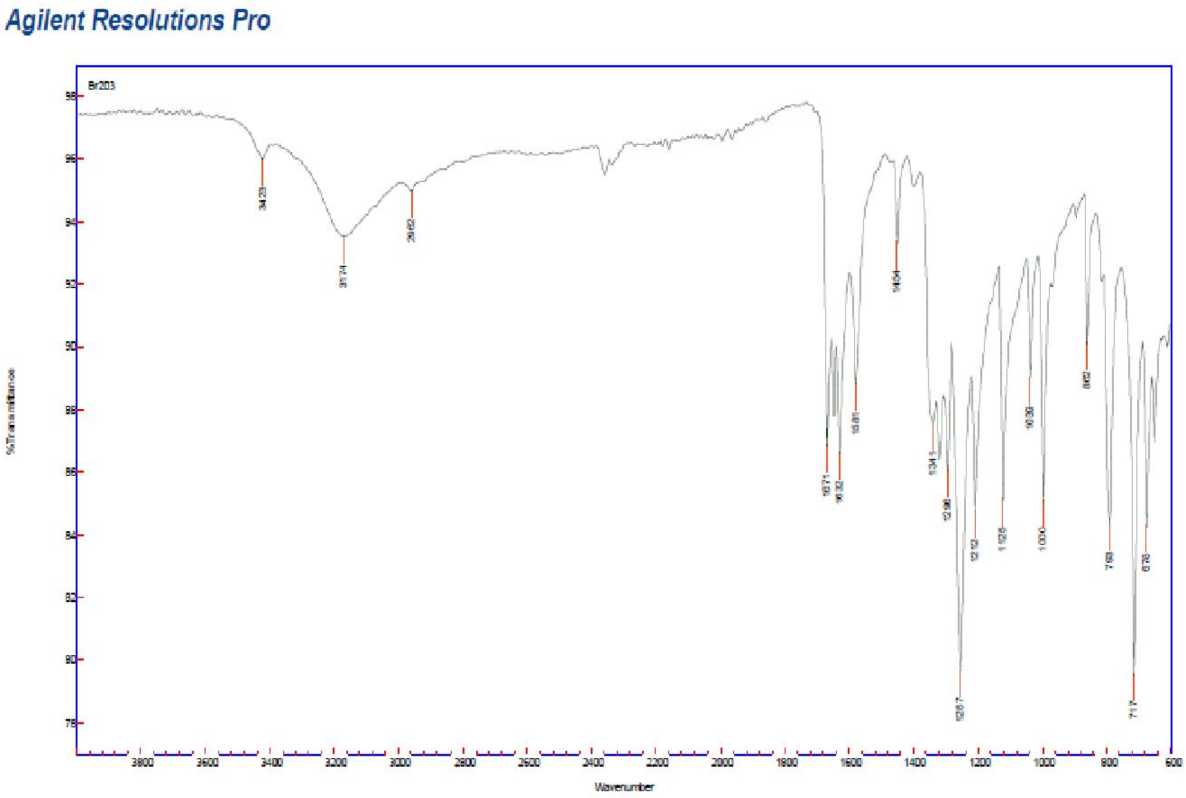

Figure S2 - Infrared spectrum of 2-bromo-3-hydroxy-1,4-naphthoquinone (**AN-02**)

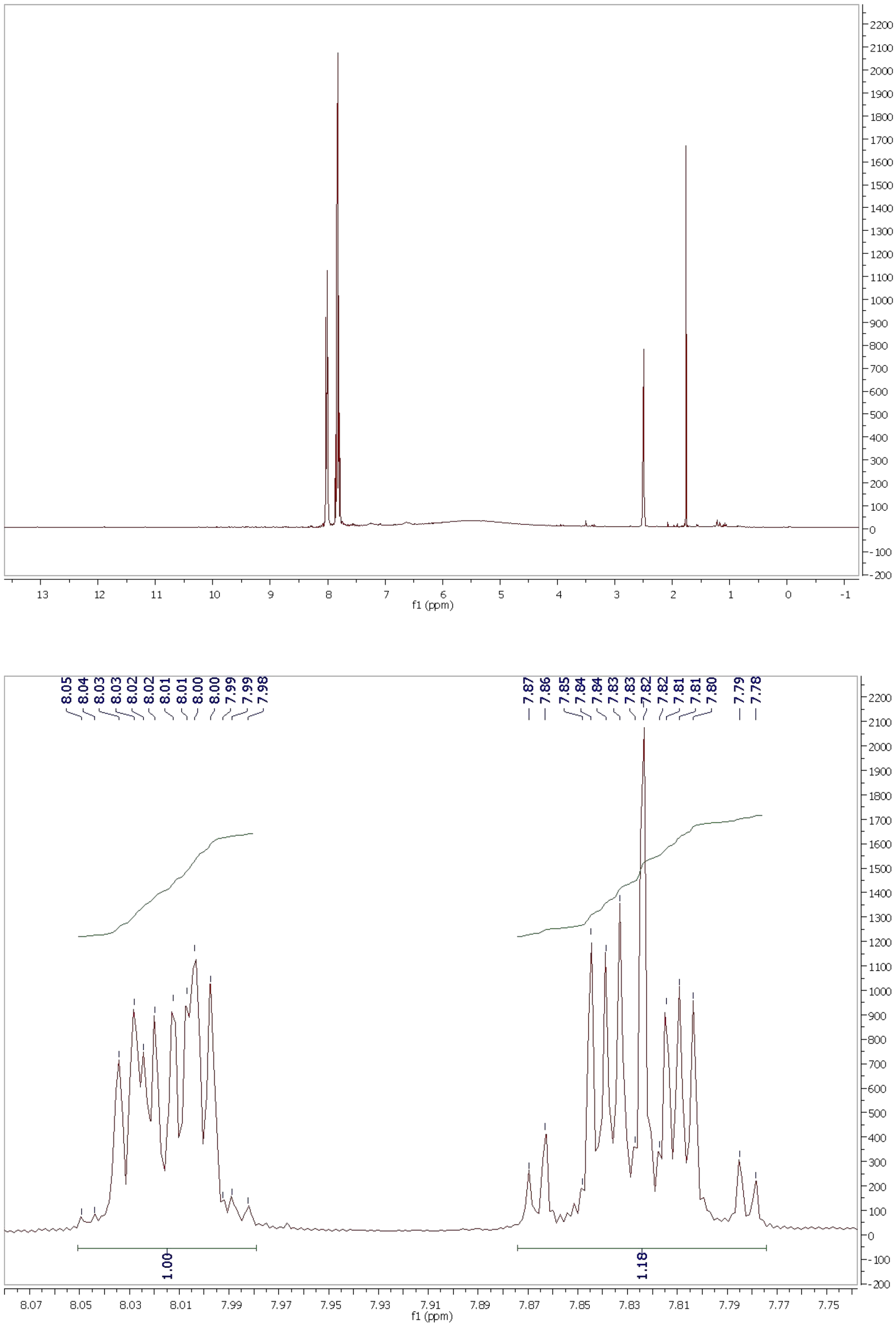

Figure S3 - ^{1}H-NMR spectrum of 2-bromo-3-hydroxy-1,4-naphthoquinone (**AN-02**)

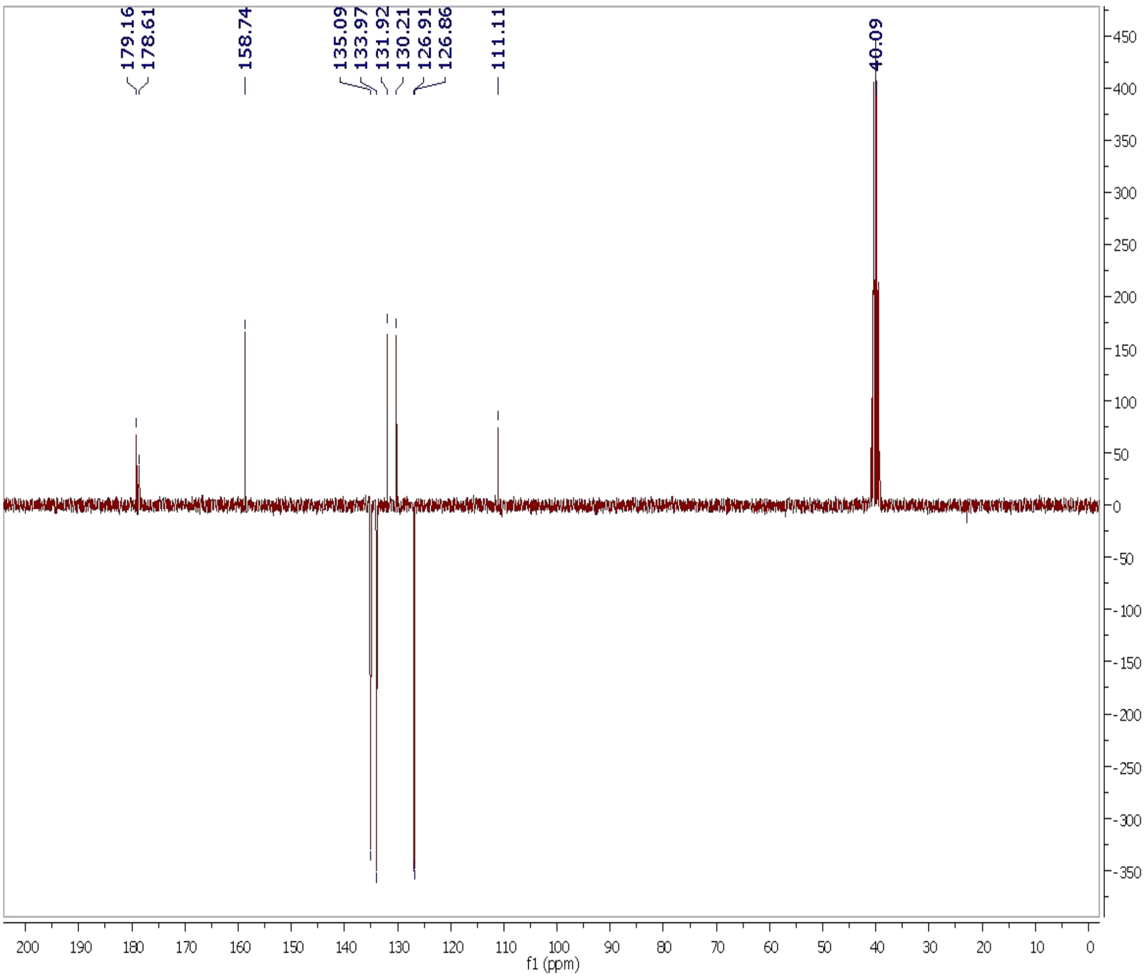

Figure S4 - ^{13}C-NMR spectrum of 2-bromo-3-hydroxy-1,4-naphthoquinone (**AN02**)

AN-03

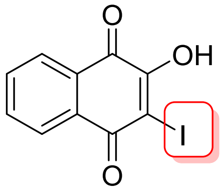

2-hydroxy-3-iodo-1,4-naphthoquinone (3-iodolawsone or 2-hydroxy-3-iodonaphthalen-1,4-dione)

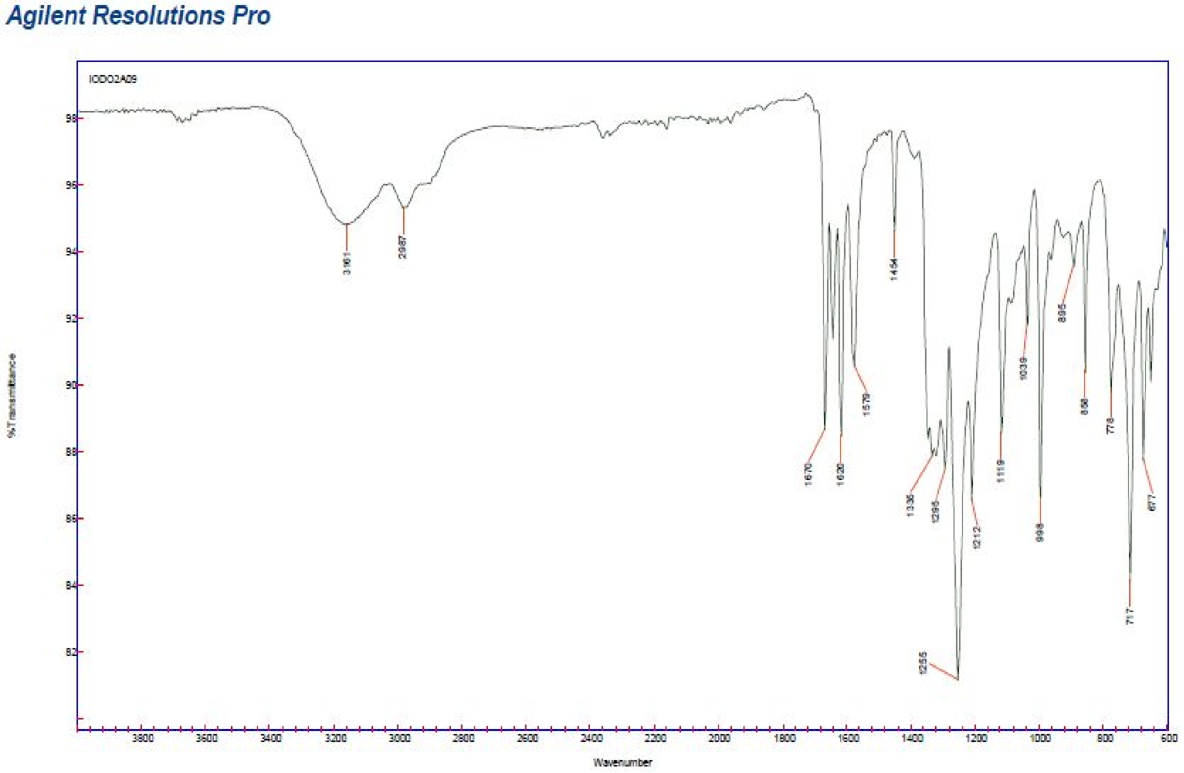

Figure S5 - Infrared spectrum of 2-hydroxy-3-iodo-1,4-naphthoquinone (**AN-03**)

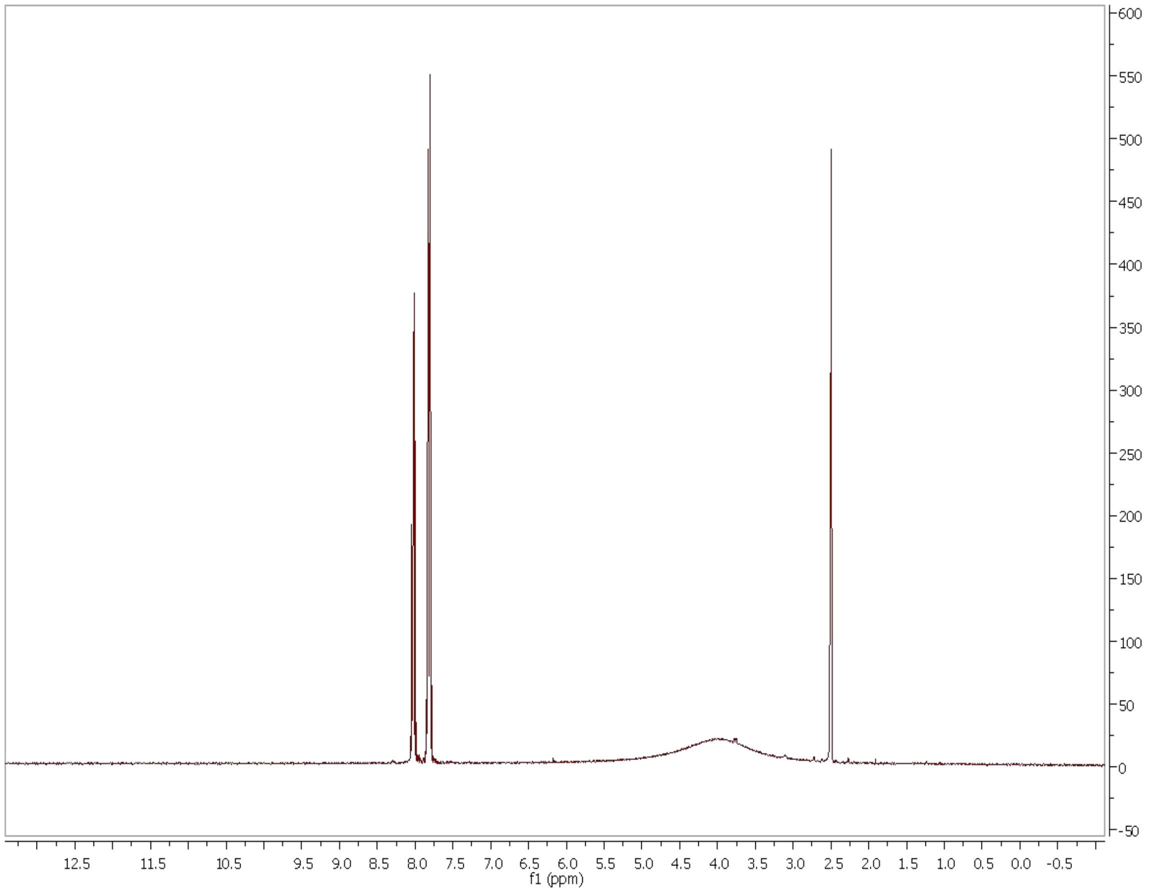

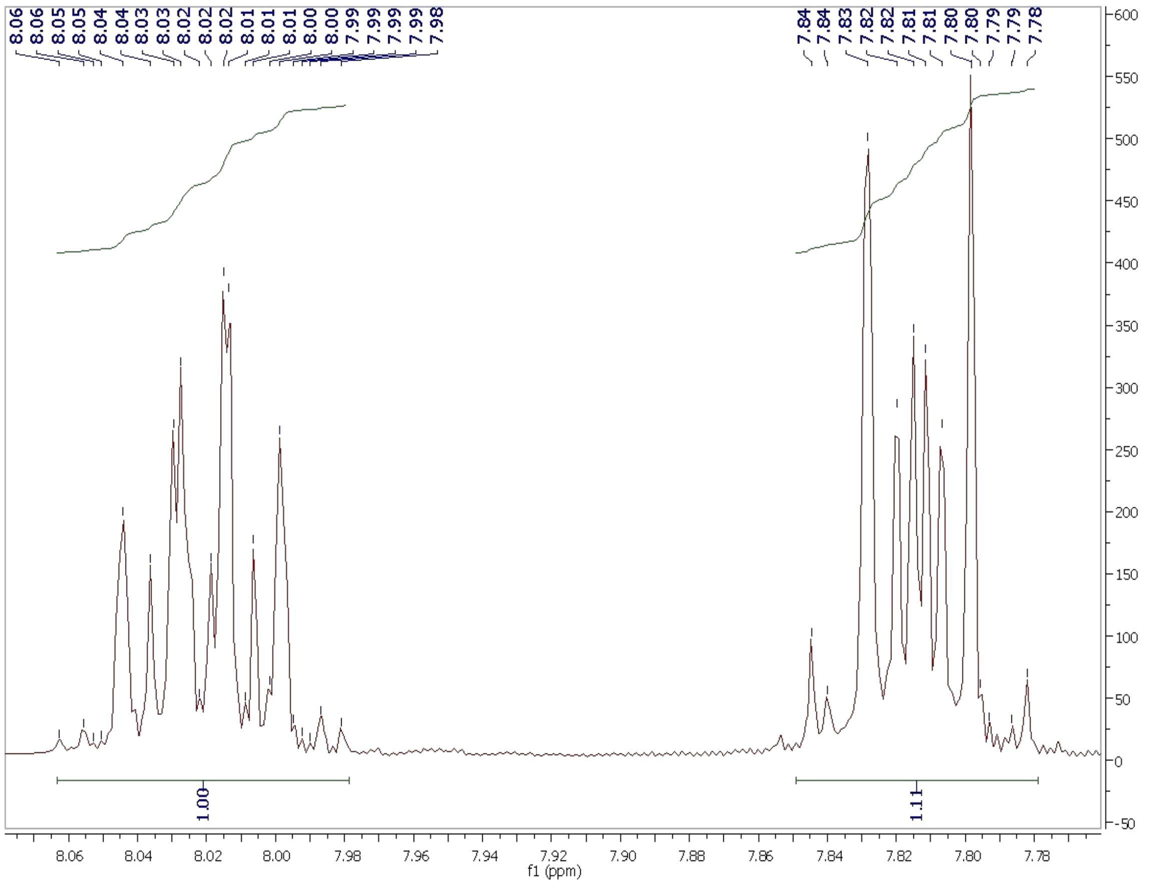

Figure S6 - ^{1}H-NMR spectrum of 2-hydroxy-3-iodo-1,4-naphthoquinone (**AN-03**)

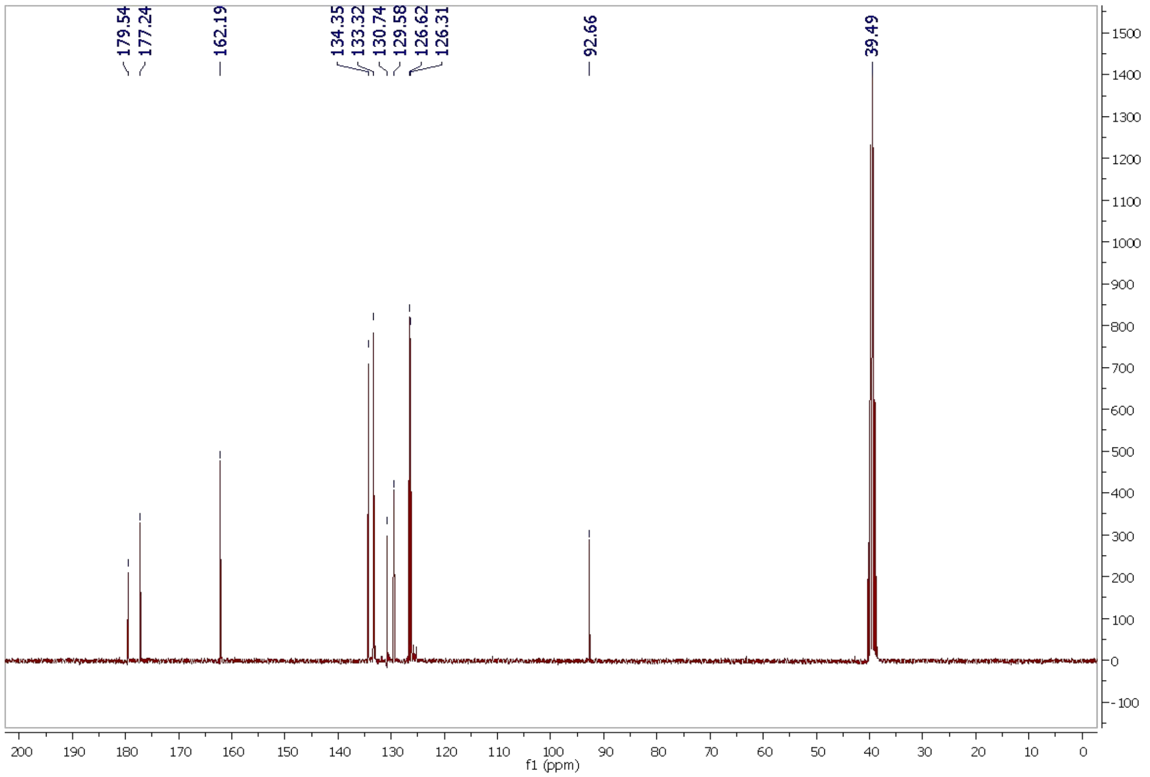

Figure S7 - ^{13}C NMR spectrum of 2-hydroxy-3-iodo-1,4-naphthoquinone **(AN-03)**

AN-04

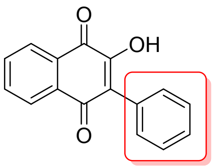

2-hydroxy-3-phenyl-1,4-naphthoquinone (2-hydroxy-3-phenylnaphthalen-1,4-dione)

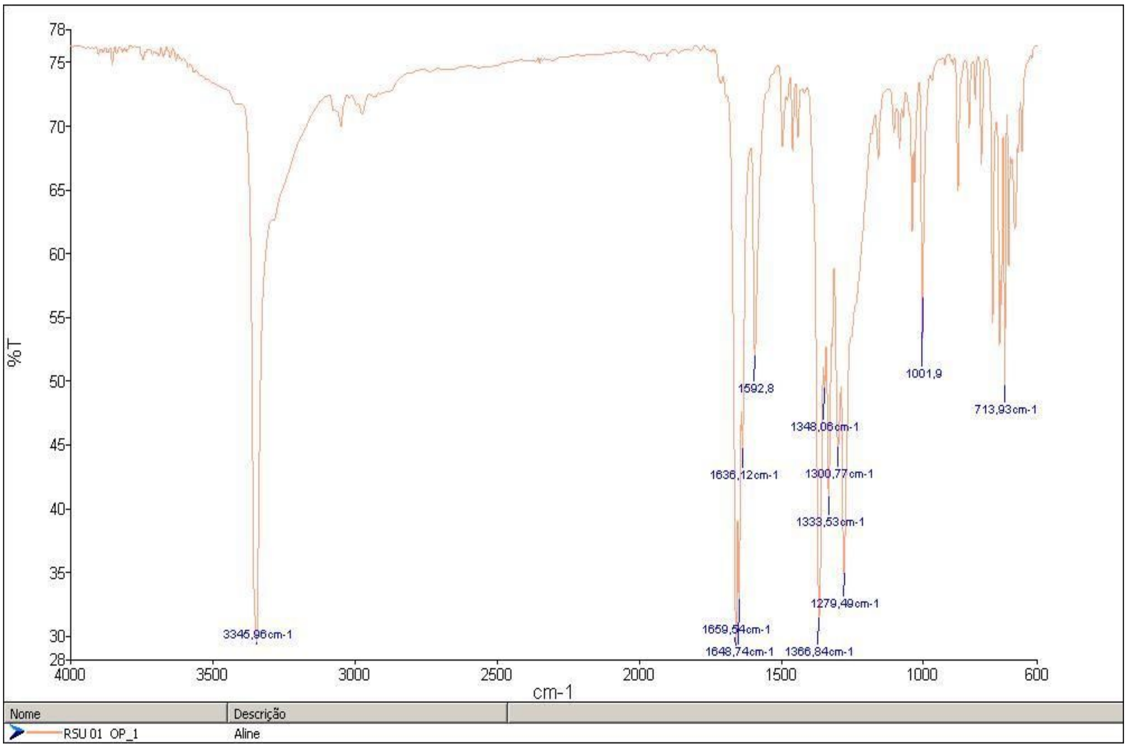

Figure S8 - Infrared spectrum of 2-hydroxy-3-phenyl-1,4-naphthoquinone (**AN-04**)

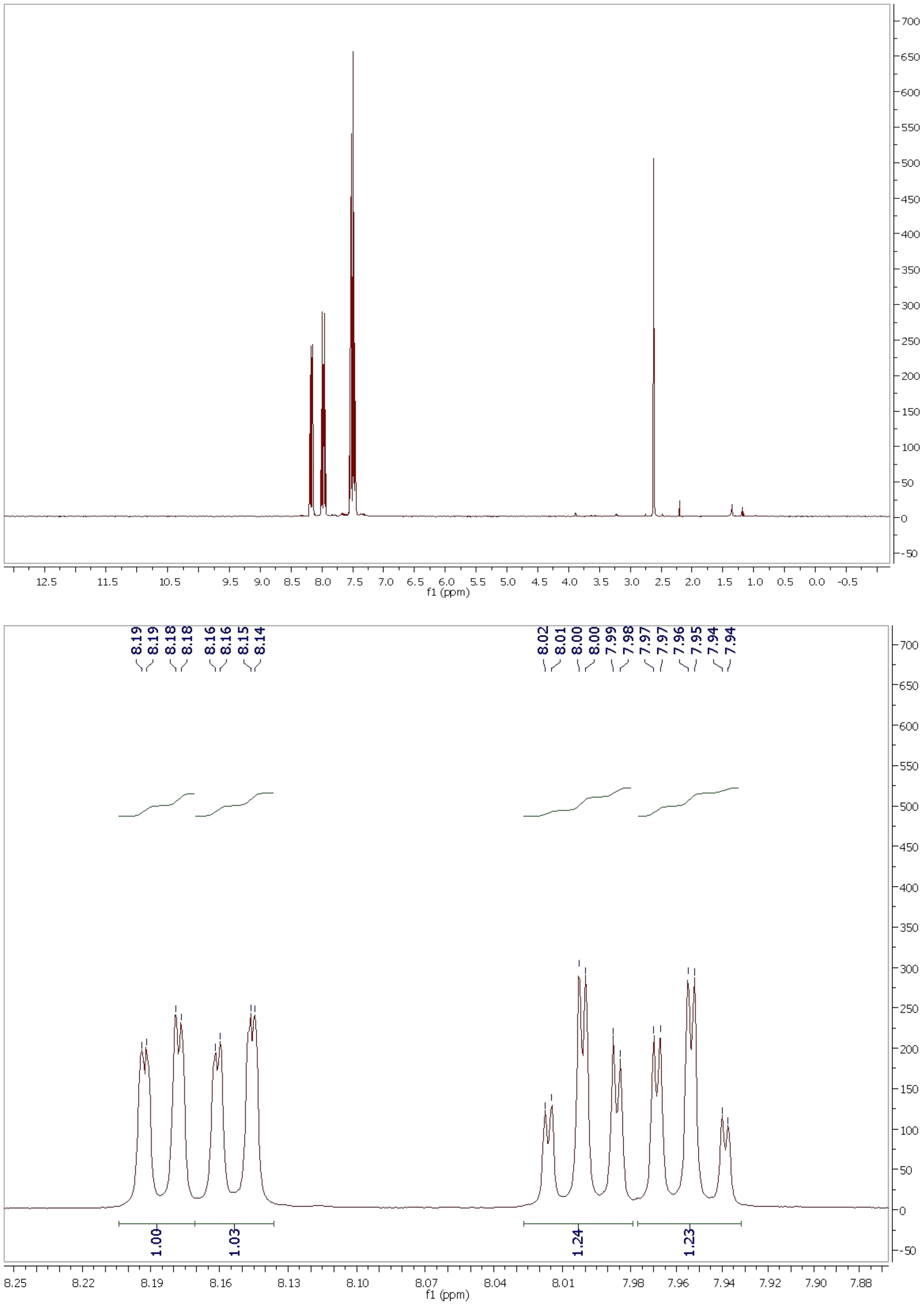

Figure S9 – ^{1}H-NMR spectrum of 2-hydroxy-3-phenyl-1,4-naphthoquinone (**AN-04**)

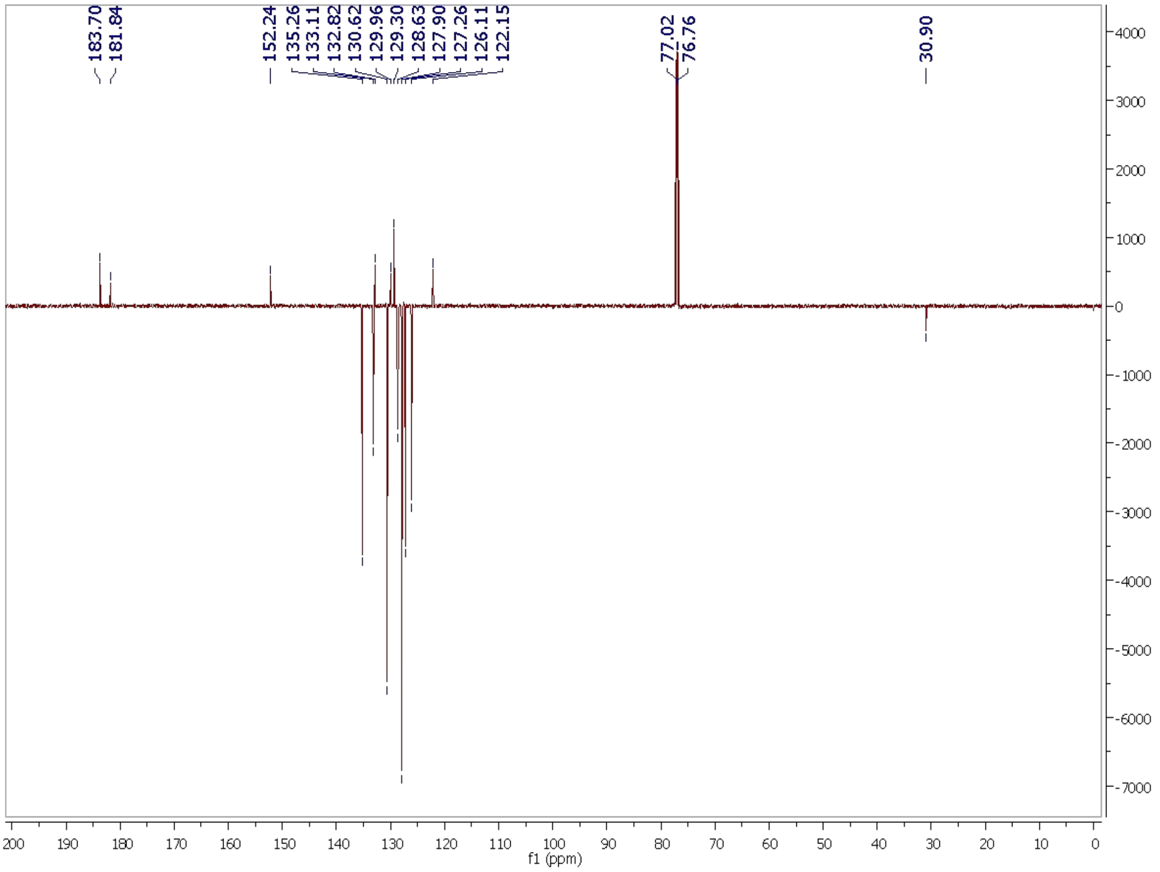

Figure S10 - ^{13}C-NMR spectrum of 2-hydroxy-3-phenyl-1,4-naphthoquinone (**AN-04**)

AN-05

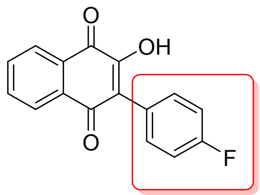

2-(4-fluorophenyl)-3-hydroxy-1,4-naphthoquinone (2-(4-fluorophenyl)-3-hydroxynaphthalen-1,4-dione)

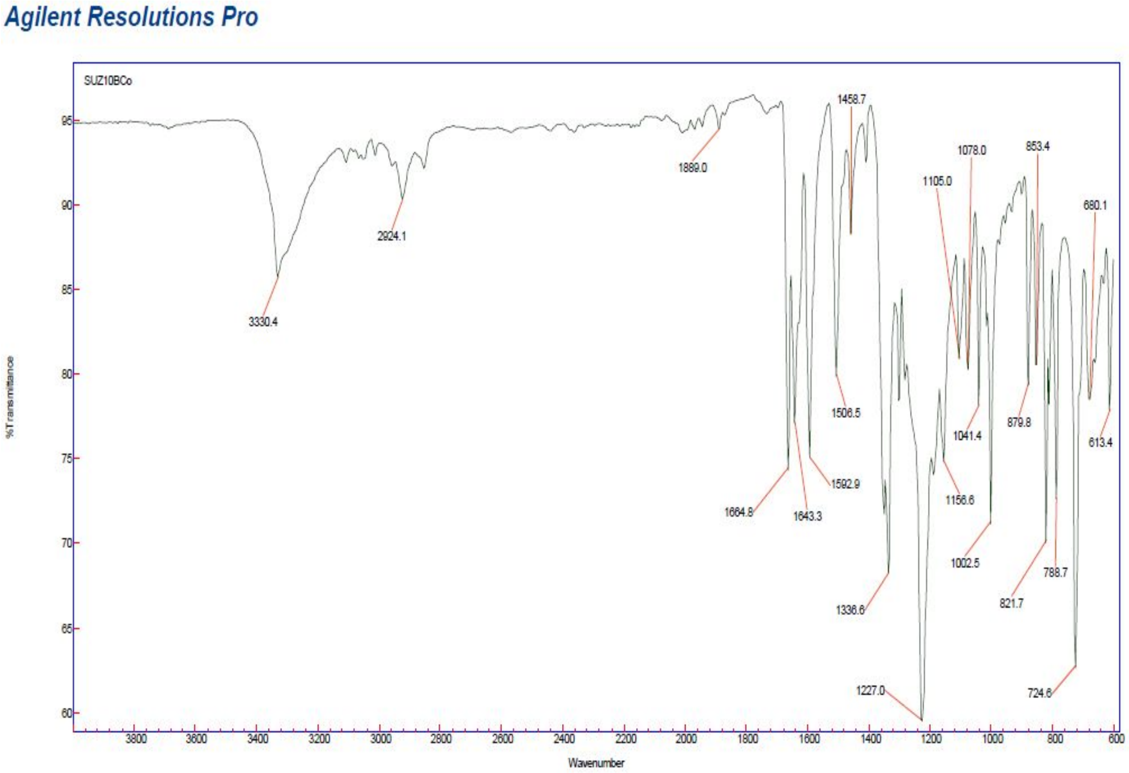

Figure S11 - Infrared spectrum of 2-(4-fluorophenyl)-3-hydroxy-1,4-naphthoquinone (**AN-05**)

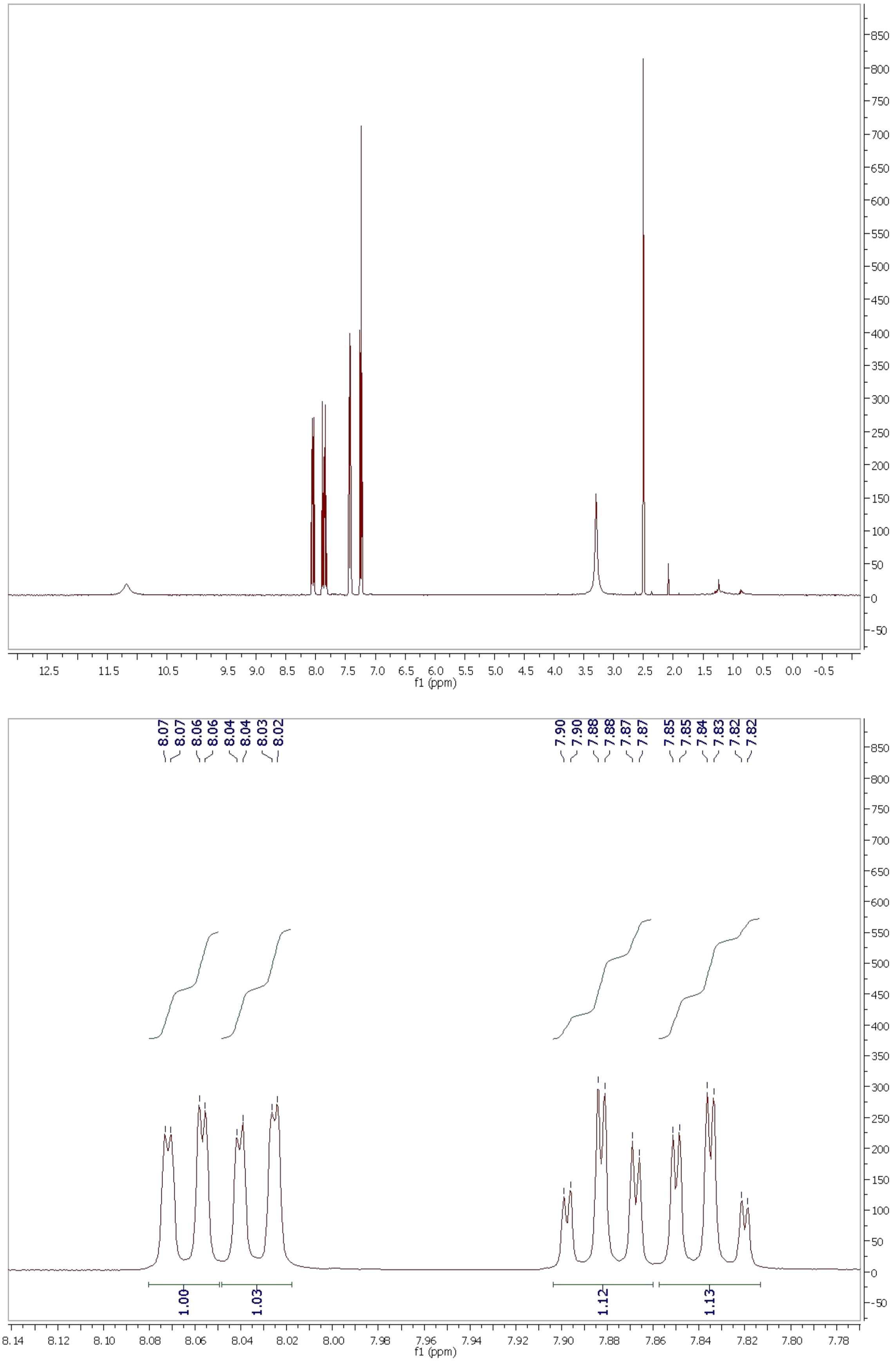

Figure S12 - ^{1}H-NMR spectrum of 2-(4-fluorophenyl)-3-hydroxy-1,4-naphthoquinone (**AN-05**)

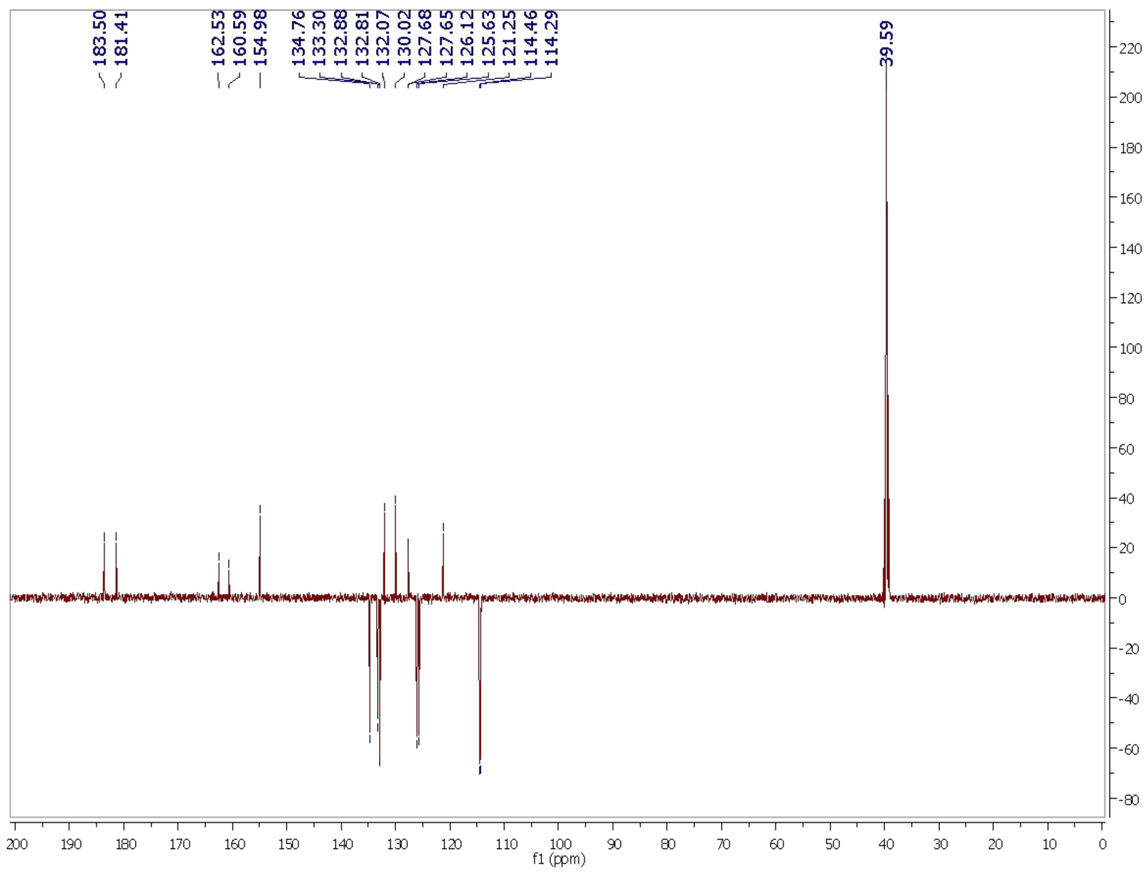

Figure S13 - ^{13}C-NMR spectra of 2-(4-fluorophenyl)-3-hydroxy-1,4-naphthoquinone (**AN-05**)

AN-06

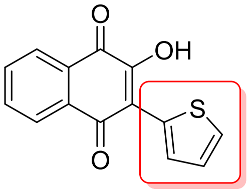

2-hydroxy-3-(thiophen-2-yl)-1,4-naphthoquinone (2-hydroxy-3-(thiophen-2-yl)naphthalen-1,4-dione)

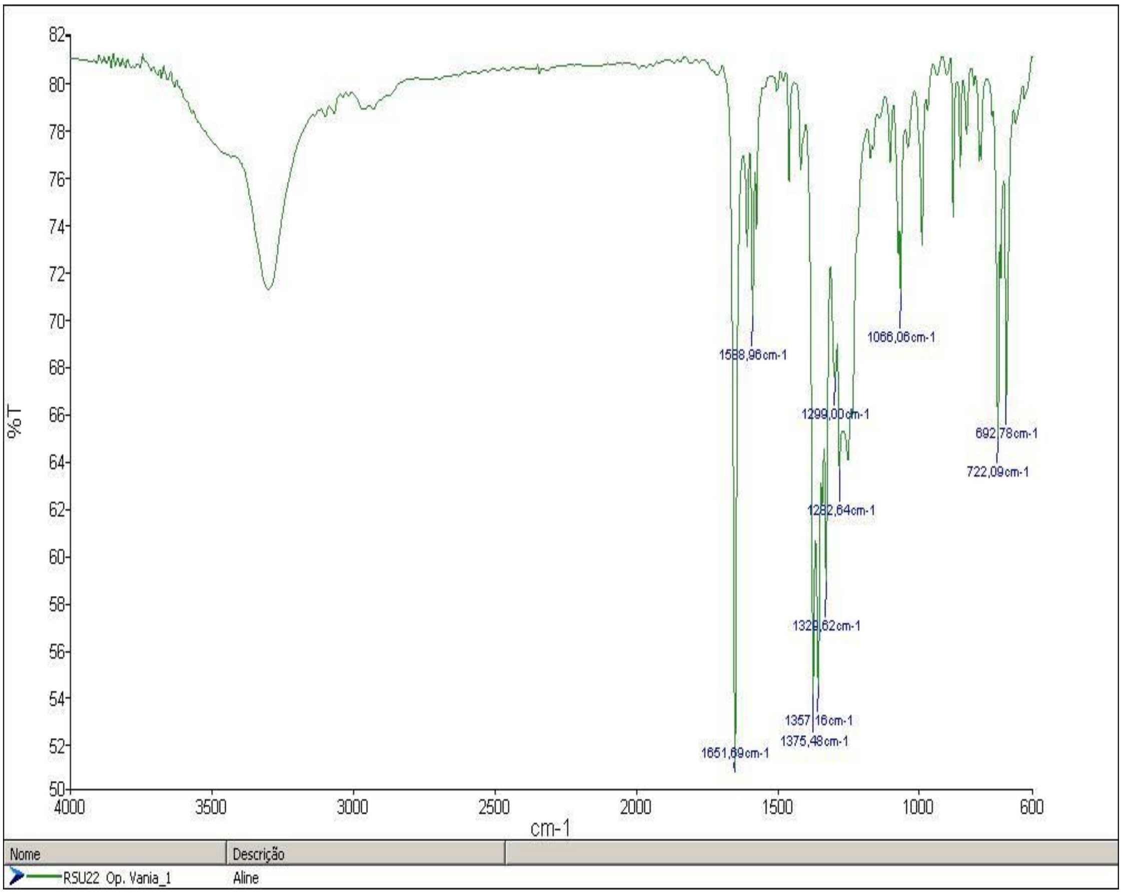

Figure S14 - Infrared spectrum of 2-hydroxy-3-(thiophen-2-yl)-1,4-naphthoquinone (**AN-06**)

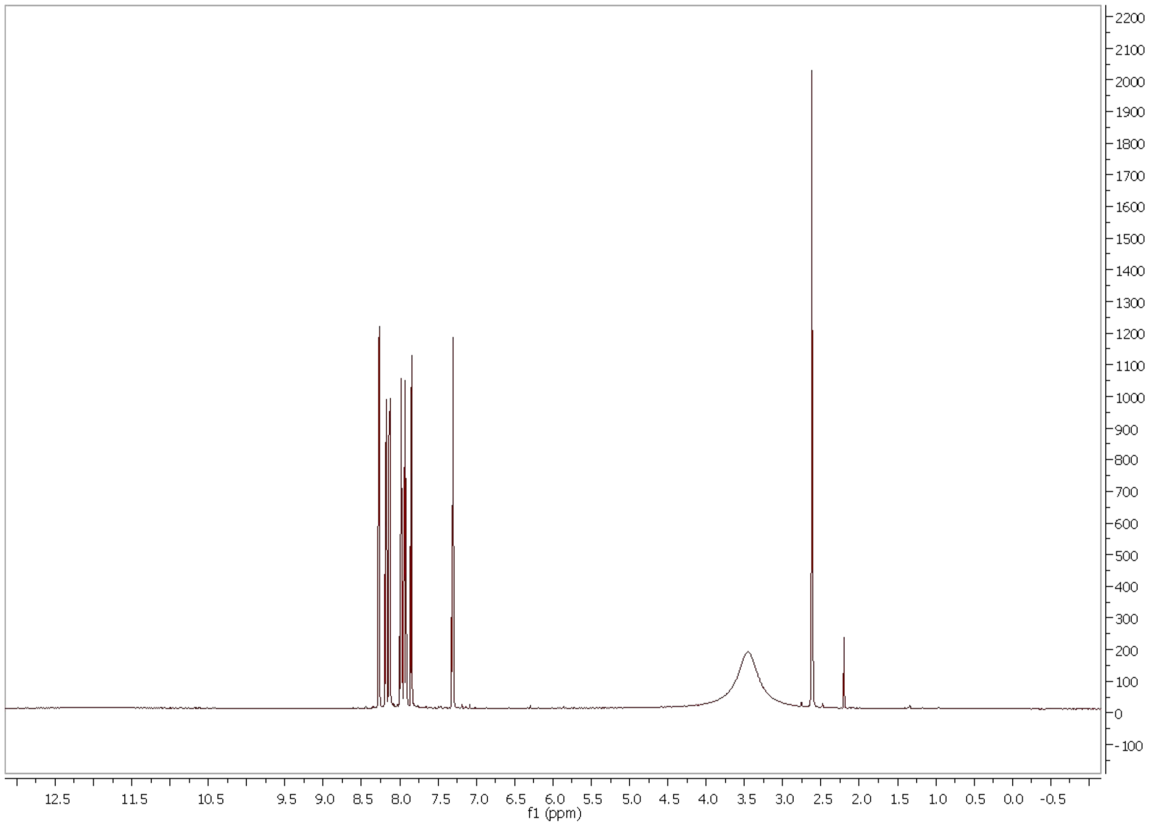
f1 (ppm)

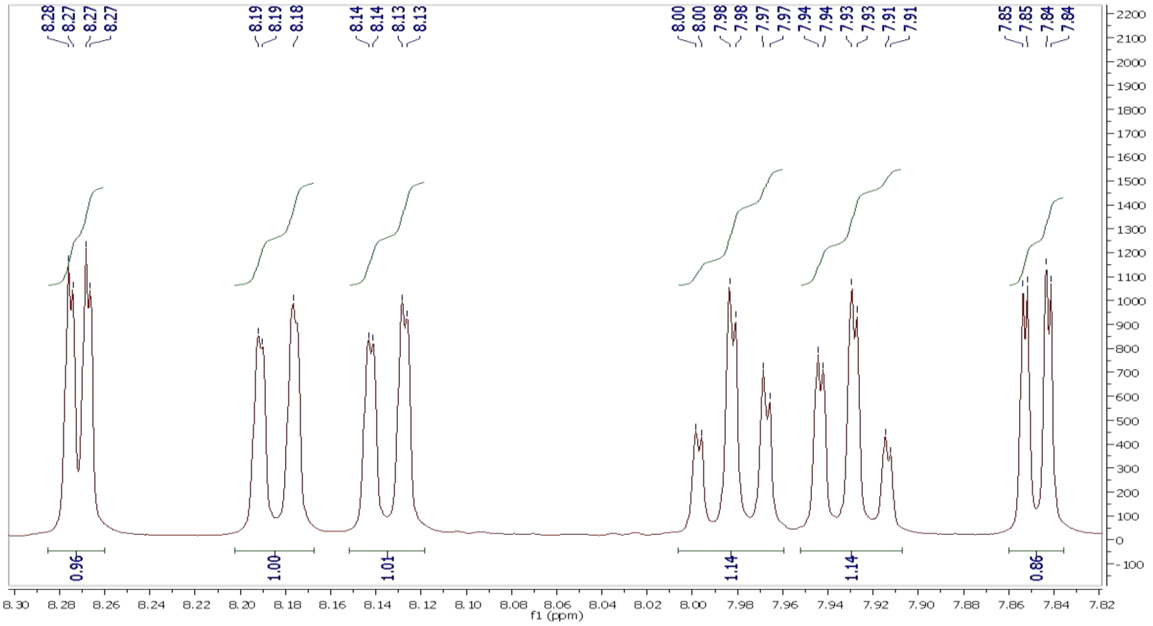
8.28
8.27
8.27
8.27
8.19
8.19
8.18
8.14
8.14
8.13
8.13
8.00
8.00
7.98
7.98
7.97
7.97
7.94
7.94
7.93
7.93
7.91
7.91
7.85
7.85
7.84
7.84
0.96
1.00
1.01
1.14
1.14
0.86
f1 (ppm)

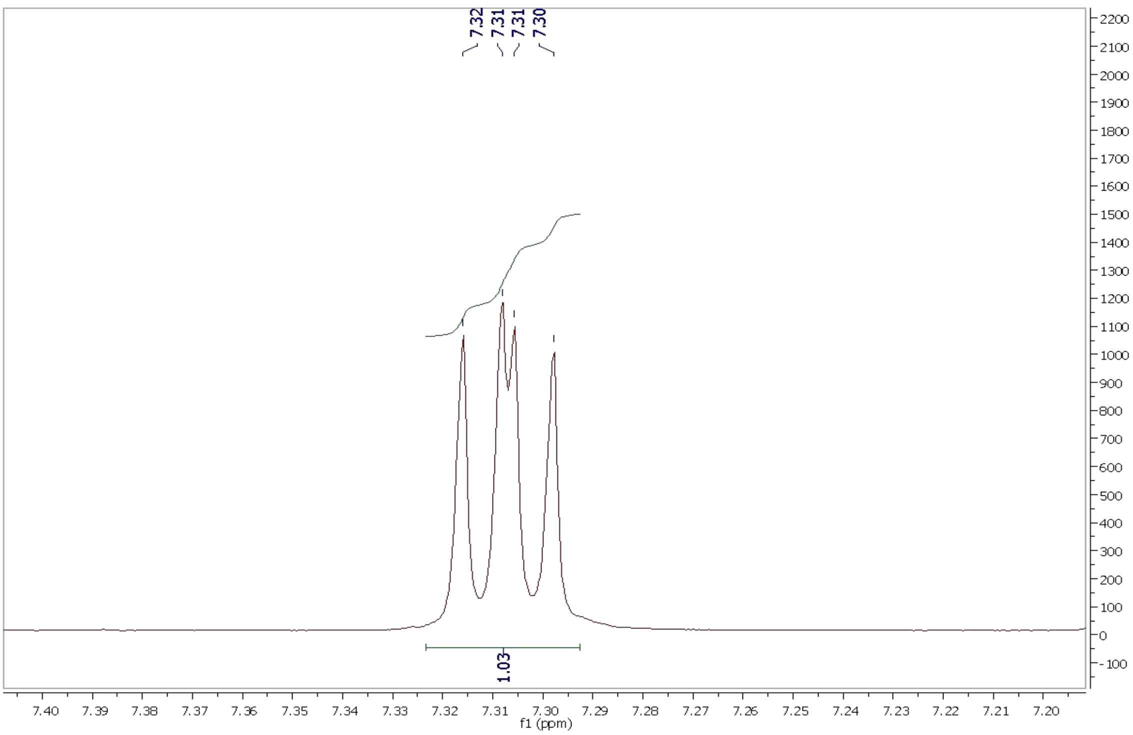

Figure S15 - ^{1}H NMR Spectra of 2-hydroxy-3-(thiophen-2-yl)-1,4-naphthoquinone (**AN-06**)

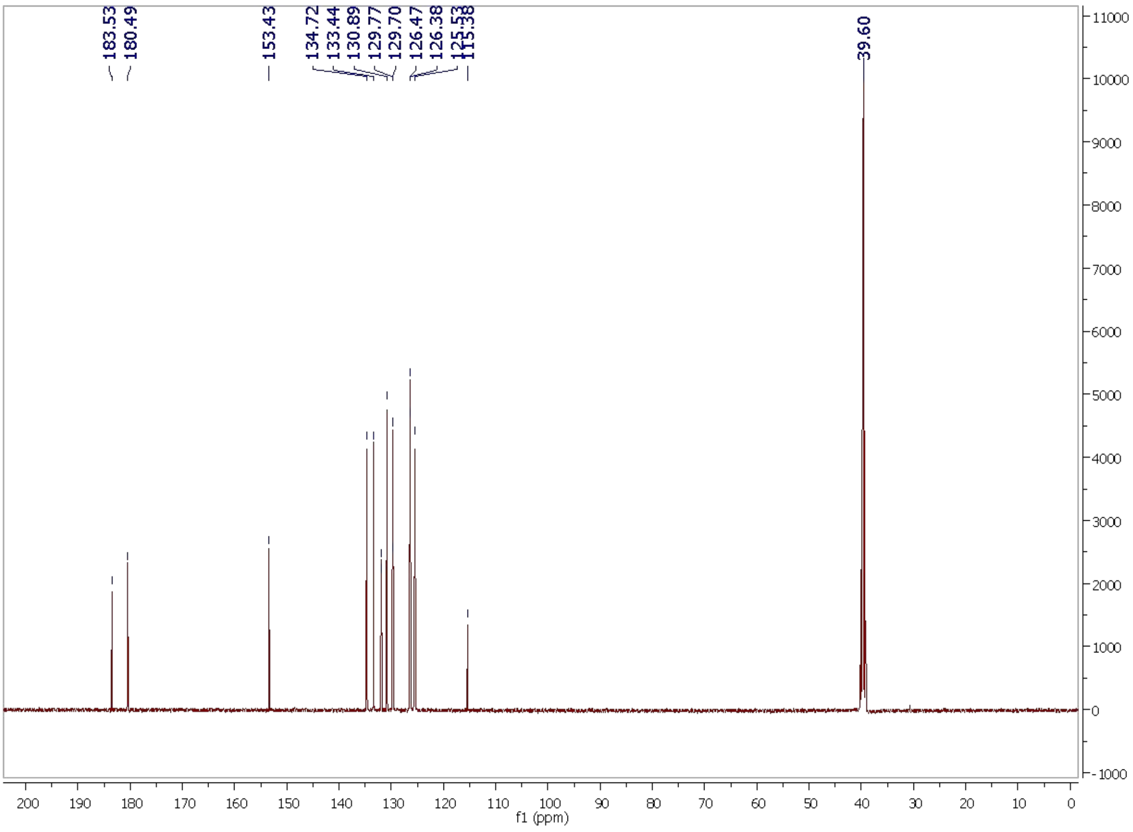

Figure S16 - ^{13}C NMR Spectra of 2-hydroxy-3-(thiophen-2-yl)-1,4-naphthoquinone (**AN-06**)

AN-07

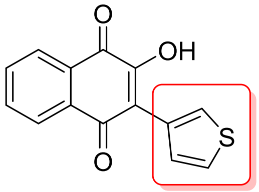

2-hydroxy-3-(thiophen-3-yl)-1,4-naphthoquinone (2-hydroxy-3-(thiophen-3-yl) naphthalen-1,4-dione)

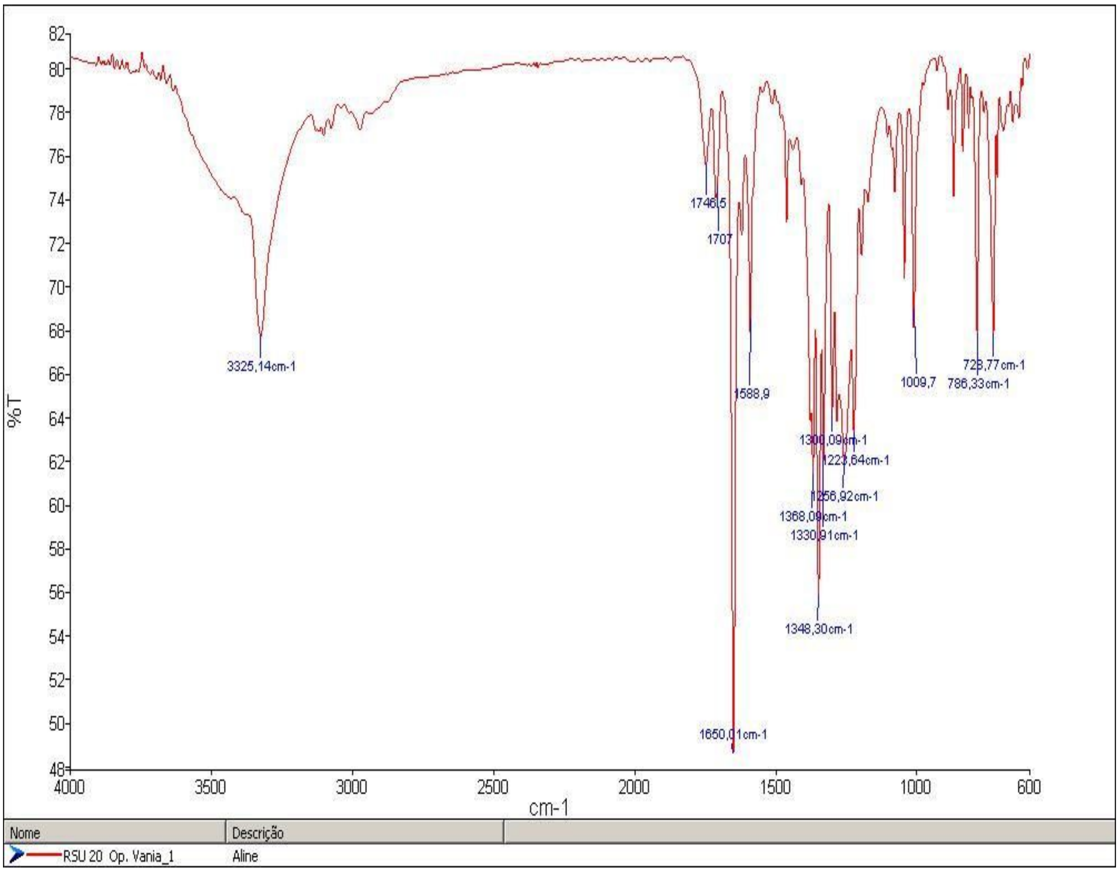

Figure S17 - Infrared spectrum of 2-hydroxy-3-(thiophen-3-yl)-1,4-naphthoquinone (**AN-07**)

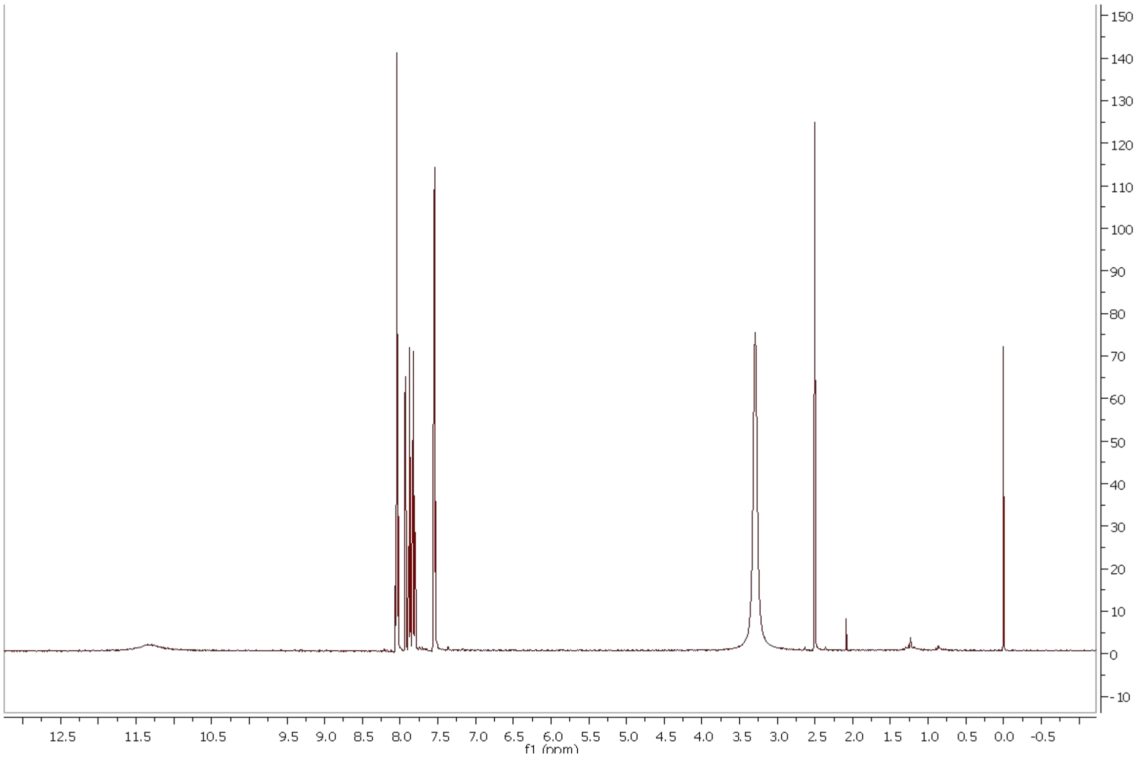

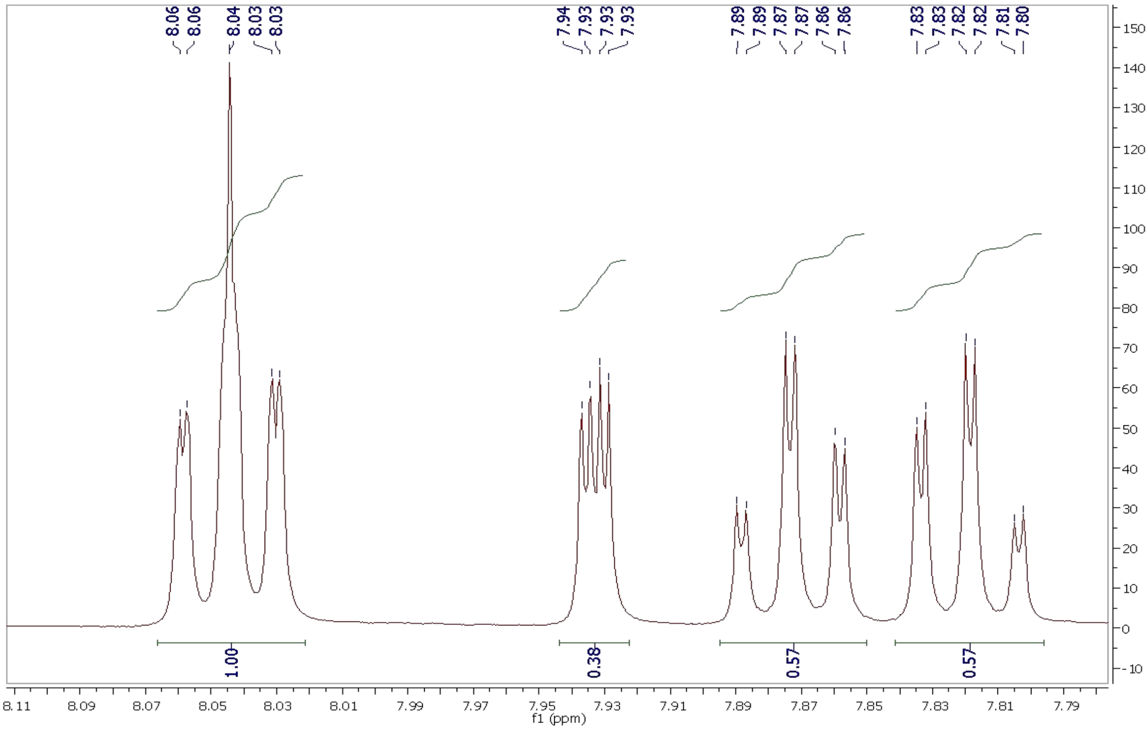

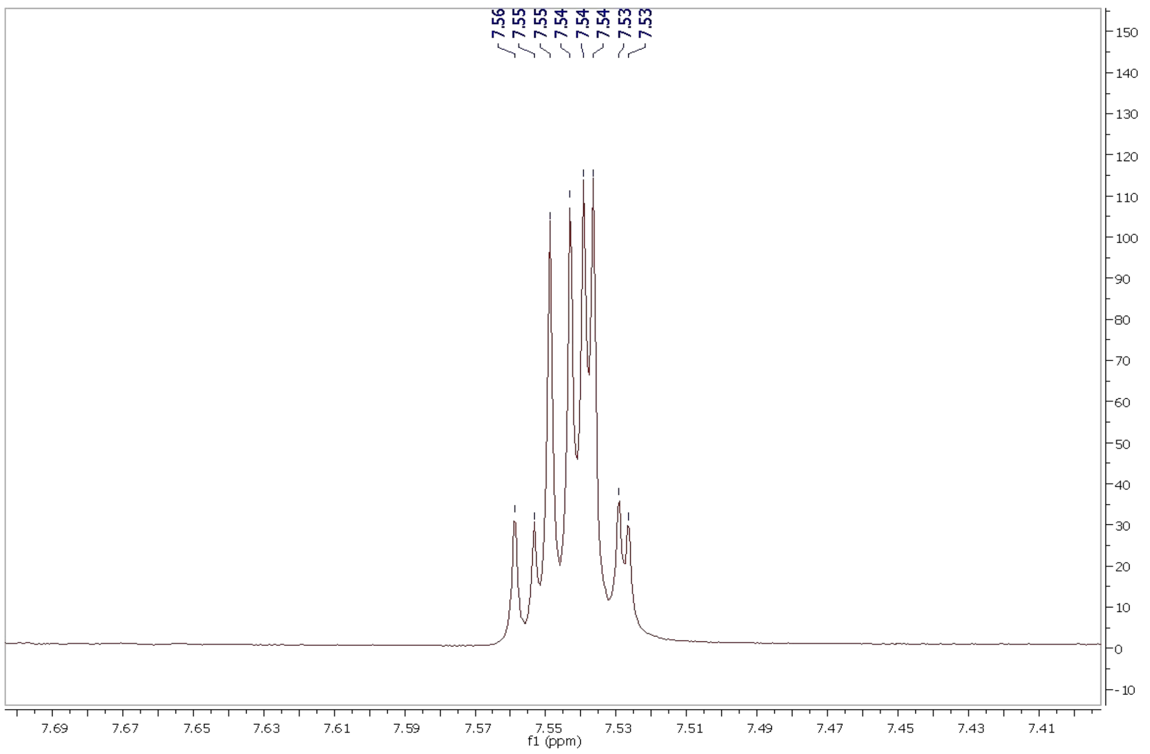

Figure S18 - ^{1}H-NMR spectrum of 2-hydroxy-3-(thiophen-3-yl)-1,4-naphthoquinone (**AN-07**)

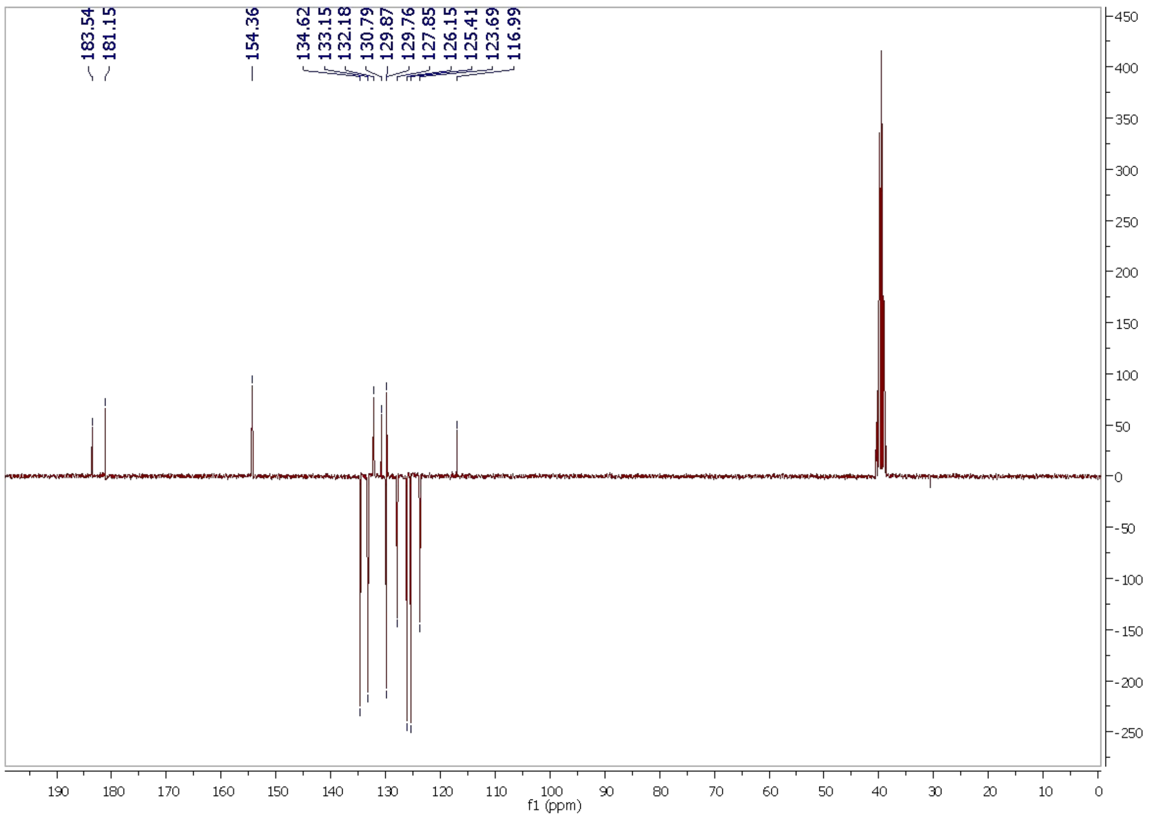

Figure S19 - ^{13}C-NMR spectrum of 2-hydroxy-3-(thiophen-3-yl)-1,4-naphthoquinone (**AN-07**)

Table S1 - Physicochemical descriptors of the naphthoquinone derivatives (AN-01-AN-07) and the anti-HSV-1 drug ACV, calculated using the FAF-Drugs4 server. MW: molecular weight; LogP: octanol-water partition coefficient; HBA and HBD: hydrogen-bond acceptor and donor groups, respectively; tPSA: topological polar surface area.

Compounds	LogP	MW (Da)	HBA	HBD	tPSA (Å²)
**AN-01**	1.38	174.15	3	1	54.37
**AN-02**	1.73	253.05	3	1	54.04
**AN-03**	1.49	300.05	3	1	54.04
**AN-04**	2.46	250.25	3	1	54.37
**AN-05**	2.56	268.24	3	1	54.37
**AN-06**	2.66	256.28	3	1	82.61
**AN-07**	2.62	256.28	3	1	82.61
**ACV**	-1.92	225.20	8	4	119.05

Table S2 - Sequence identity (%) and validation results obtained for the human NKA models in E1 (hNKA-E1) and E2 (hNKA-E2) states and their templates.

Structures	Sequence identity (%)	Ramachandran plot			% residues with score 3D1D ≥ 0.2	Z-score
		Favorable (%)	Allowed (%)	Disallowed (%)		
**hNKA-E1**	85.91	93.30	6.70	0.00	80.89	-11.35
**3WGU**		85.70	14.30	0.00	85.61	-11.61
**hNKA-E2**	84.04	93.90	6.10	0.00	84.78	-11.53
**3A3Y**		87.90	12.10	0.00	83.47	-10.80

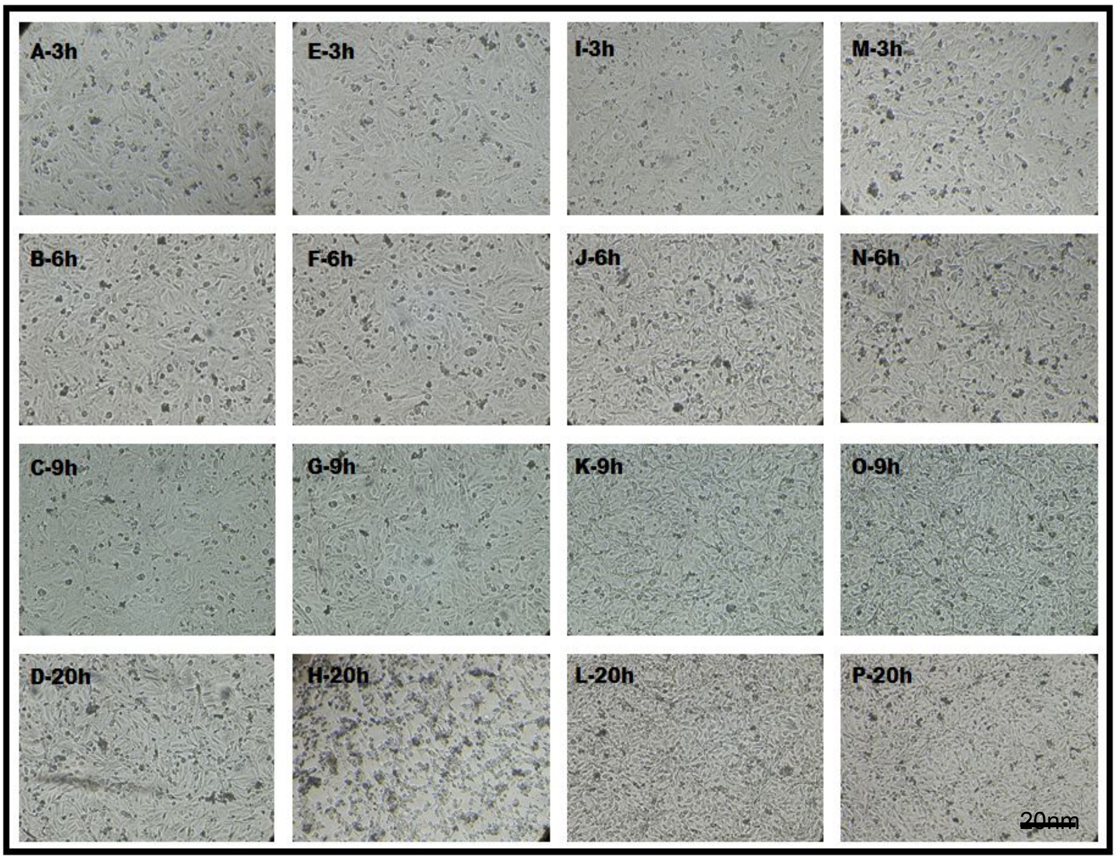

Figure S20 - Illustrative images of VERO cells infected with HSV-1 and treated with synthetic naphthoquinone AN-06 at 3 h.p.i, 6 h.p.i, 9 h.p.i and 20 h.p.i. The images were capture in a light microscope. Images A, B, C, and D - Cell control; E, F, G, and H images - Virus control; Images I, J, K, and L - Infected and treated with AN-06; M, N, O and P - Treated with AN-06. All cells are in Hank's solution. 400x magnification in the bright field.
